# Compensatory Mitophagy via NEDD4/HIF‐1α/BNIP3 Pathway Restrains EndMT and Renal Allograft Interstitial Fibrosis Induced by TNFα

**DOI:** 10.1002/advs.202516953

**Published:** 2026-09-20

**Authors:** Dengyuan Feng, Qinghuan Shen, Junqi Zhang, Jiawen Liu, Zeping Gui, Zijie Wang, Qianguang Han, Shuang Fei, Hao Chen, Li Sun, Jun Tao, Zhijian Han, Xiaobing Ju, Min Gu, Jianjian Zhang, Ruoyun Tan

**Affiliations:** ^1^ Department of Urology the First Affiliated Hospital with Nanjing Medical University Nanjing China; ^2^ Department of Andrology, Nanjing Drum Tower Hospital Affiliated Hospital of Medical School Nanjing China; ^3^ Department of Urology the Second Affiliated Hospital of Nanjing Medical University Nanjing China

**Keywords:** EndMT, mitophagy, NEDD4, renal allograft interstitial fibrosis, TNFα

## Abstract

Renal allograft interstitial fibrosis/tubular atrophy (IF/TA) is the pathological hallmark of chronic renal allograft dysfunction (CAD) and endothelial‐to‐mesenchymal transition (EndMT) has been implicated as one of the key triggering factors. Here, we identified tumor necrosis factor‐α (TNFα) as a key inducer of mitochondrial dysfunction and EndMT in endothelial cells of renal allografts. Importantly, TNFα simultaneously activated BNIP3‐dependent mitophagy as a compensatory response, which removed damaged mitochondria, restrained EndMT, and attenuated renal allograft interstitial fibrosis. In addition, inhibition of BNIP3 promoted the accumulation of damaged mitochondria, aggravated EndMT, and exacerbated renal allograft interstitial fibrosis, supporting a protective role of mitophagy. Mechanistically, TNFα upregulated and stabilized HIF‐1α by disrupting its NEDD4‐mediated ubiquitination, thereby enhancing BNIP3 transcription to drive mitophagy. Moreover, endothelial‐specific deletion of HIF‐1α impaired this compensatory pathway, aggravated mitochondrial dysfunction, and exacerbated renal allograft interstitial fibrosis. Thus, compensatory mitophagy through the NEDD4/HIF‐1α/BNIP3 pathway represented a potential protective stress‐adaptation program in renal allograft endothelial cells, providing mechanistic insight and a conceptual framework for future therapeutic exploration aimed at preserving renal allograft function.

## Graphical abstract

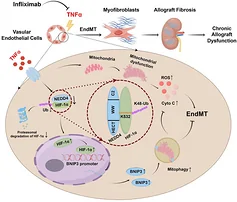

## 1 Introduction

Kidney transplantation remains the optimal treatment for patients with end‐stage renal disease [1]. However, long‐term renal allograft survival is limited by chronic renal allograft dysfunction (CAD), which is primarily manifested as renal allograft interstitial fibrosis and tubular atrophy (IF/TA) [2, 3]. Endothelial‐to‐mesenchymal transition (EndMT), in which vascular endothelial cells transdifferentiate into matrix‐producing myofibroblasts in response to immunological and non‐immunological insults, has been implicated as one of the key triggering factors for IF/TA in CAD [4]. Inflammatory mediators are well‐established contributors to kidney fibrosis [5, 6], yet the mechanisms underlying EndMT in CAD remain incompletely understood. A detailed understanding of the molecular pathways regulating EndMT is therefore essential for elucidating the pathogenesis of renal allograft interstitial fibrosis and informing future therapeutic exploration.

Oxidative stress, largely driven by mitochondrial dysfunction, is a central mediator of kidney fibrosis [7]. Mitochondria are essential for cellular homeostasis, providing energy and maintaining redox balance [8], yet mitochondrial impairment contributes directly to renal fibrogenesis [9]. Dysfunctional mitochondria reduce adenosine triphosphate (ATP) production, elevate reactive oxygen species (ROS), and amplify pro‐fibrotic signaling [10], thereby promoting inflammation and extracellular matrix (ECM) deposition [9]. Moreover, mitochondrial dysfunction and ROS accumulation are closely linked to EndMT [11, 12], and ROS scavengers can mitigate EndMT, highlighting the critical role of mitochondrial integrity in endothelial homeostasis [13, 14]. Despite these insights, the mechanisms connecting vascular endothelial mitochondrial dysfunction to renal allograft interstitial fibrosis remain incompletely understood.

Mitophagy, the selective autophagic removal of damaged mitochondria, is critical for maintaining mitochondrial quality and cellular homeostasis [15]. It occurs primarily via receptor‐mediated or ubiquitin‐mediated pathways [16]. In the progression of kidney fibrosis, mitophagy is increasingly recognized as a protective mechanism that counteracts mitochondrial dysfunction and fibrogenesis [17, 18]. For instance, Li et al. showed that mitophagy attenuates kidney fibrosis by reducing ROS generation and suppressing NOD‐like receptor family pyrin domain containing 3 (NLRP3) inflammasome activation [19]. Nevertheless, its role in renal allograft interstitial fibrosis remains poorly defined. Moreover, the relationship between TNFα and mitophagy is context‐dependent, with conflicting reports across disease models [20, 21], highlighting the need to clarify the molecular mechanisms linking TNFα and mitophagy in renal allograft fibrosis.

The neuronally expressed developmentally downregulated 4 (NEDD4) family of E3 ubiquitin ligases is characterized by a C2‐WW(n)‐HECT domain architecture and plays a critical role in human physiology and pathophysiology [22]. Among them, NEDD4 (also known as NEDD4‐1) regulates cellular growth and proliferation and is essential for neuronal development and function [23].

In this study, we delineated the regulatory role and underlying mechanism of tumor necrosis factor‐α (TNFα) in endothelial cells during renal allograft interstitial fibrosis. We found that the role of TNFα in this process was complex and multifaceted. In endothelial cells, TNFα promoted renal allograft interstitial fibrosis by impairing mitochondrial function and inducing EndMT formation. However, a compensatory mitophagy induced by TNFα could clear damaged mitochondria and inhibit EndMT. Mechanistically, TNFα inhibited proteasomal degradation of hypoxia‐inducible factor‐1α (HIF‐1α) in endothelial cells by disrupting the interaction between HIF‐1α and the E3 ubiquitin ligase NEDD4. Subsequently, stabilized HIF‐1α promoted transcription of BCL2/adenovirus E1B 19 kDa interacting protein 3 (BNIP3), and activated BNIP3‐mediated mitophagy. This NEDD4–HIF‐1α–BNIP3–mediated mitophagy pathway functioned as a compensatory mechanism in this setting to protect against the progression of interstitial fibrosis in transplanted kidneys.

## 2 Results

### 2.1 TNFα is Elevated and Associated With EndMT and Renal Allograft Interstitial Fibrosis

Endothelial cells are a crucial component of renal tissue and serve as the primary interface between the recipient and donor, making them highly susceptible to both immune‐ and non‐immune–mediated injuries after kidney transplantation. EndMT has been established as one of the key contributors to renal allograft fibrosis [24]. To identify critical drivers of EndMT, we systematically analyzed the publicly available GSE195718 dataset. The dataset characteristics are summarized in Figure S1. Patients were stratified into two groups: a normal allograft group (renal allograft tissues from three recipients with stable kidney function, Normal group) and a fibrotic allograft group (renal allograft tissues from six recipients with CAD, CAD group). Uniform Manifold Approximation and Projection (UMAP) analyses identified major kidney cell types using lineage‐specific markers (Figure 1A), with endothelial cells defined by CD31 and CD34 expression (Figure 1B). GSEA of DEGs revealed significant enrichment of inflammatory response and TNF signaling, suggesting that inflammatory mediators play a pivotal role in renal allograft fibrosis (Figure 1C). Based on previous studies demonstrating that TNFα, interleukin (IL)‐1β, and IL‐6 are key inflammatory cytokines involved in inflammatory signaling [25, 26], we next measured serum levels of these cytokines in kidney transplant recipients. Among them, TNFα showed the most significant increase (Figure 1D). Elevated TNFα expression in CAD specimens was further validated through histological analyses. Compared with the Normal group, CAD tissues displayed severe interstitial fibrosis and tubular injury (Figure 1E). Moreover, evidence of EndMT in CAD tissues was indicated by increased FN, decreased CD31, and elevated TNFα expression in immunohistochemical (IHC) staining assays (Figure 1F). Importantly, microvessel densities, identified by the endothelial marker CD31, were significantly reduced in CAD tissues (Figure 1G). To validate our findings from human specimens, we employed a murine renal allogeneic transplantation model. Syngeneic controls (Syn; BALB/c recipients of BALB/c kidneys) were compared with allogeneic transplants (Allo; C57BL/6 recipients of BALB/c kidneys). Serum TNFα levels were significantly higher in Allo mice compared with Syn controls (Figure 1H). Histological analyses using Periodic Acid‐Schiff (PAS) and Masson staining assays revealed pronounced tubular injury, glomerulosclerosis, and interstitial fibrosis in Allo allografts (Figure 1I). Consistent with these findings, immunofluorescence (IF) staining assays demonstrated increased colocalization of CD31 and αSMA in Allo allograft tissues (Figure 1J). WB assays further confirmed the loss of endothelial markers (EMCN and CD31) and concomitant upregulation of mesenchymal markers (αSMA and Collagen I), indicative of EndMT (Figure 1M). In addition, microvessel densities were significantly reduced in allograft tissues from the Allo group (Figure 1K). In parallel, TNFα upregulation in allograft tissues from Allo mice was validated by Western blot (WB) analyses (Figure 1N). Pearson's correlation analysis revealed a strong negative correlation between TNFα and CD31 expression (R^2^ = 0.9378, *p* < 0.001), and positive correlation with FN expression (R^2^ = 0.9921, *p* < 0.001) (Figure 1L). In vitro studies using human umbilical vein endothelial cells (HUVECs) further substantiated these observations. TNFα stimulation led to a marked reduction in CD31 expression, accompanied by increased αSMA and FN levels, consistent with EndMT (Figure 4A,B). Collectively, these findings suggest that TNFα is associated with EndMT and renal allograft interstitial fibrosis following kidney transplantation.

**FIGURE 1:**
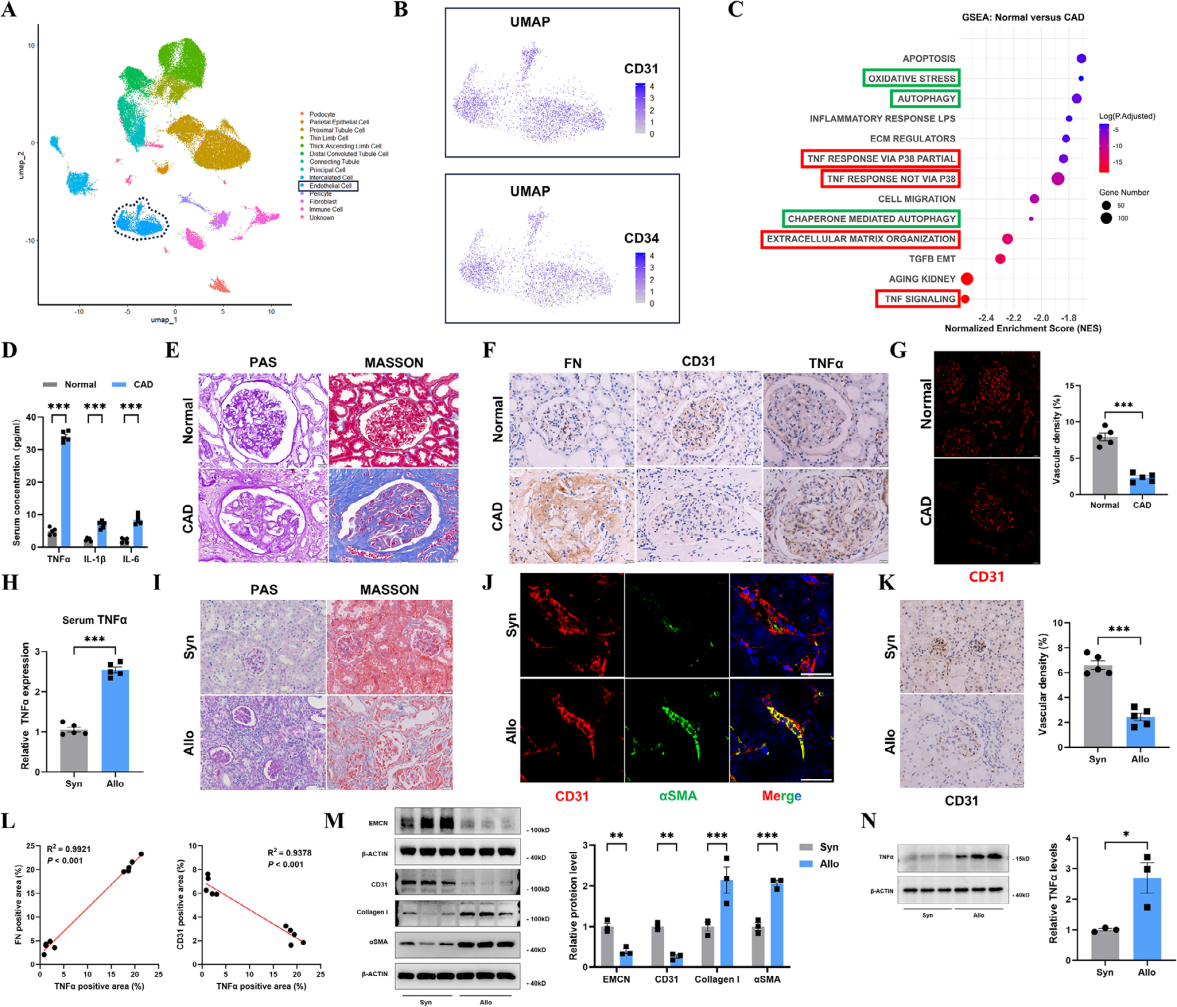
TNFα is a prominent inflammatory cytokine associated with renal allograft interstitial fibrosis. (A) UMAP visualization of cell subclusters in the GSE195718 dataset. Different subclusters are color‐coded. (B) UMAPs display the expression of classical endothelial cell markers, CD31 and CD34. (C) GSEA of DEGs in endothelial cells between the Normal and CAD group recipients. (D) Serum levels of TNFα, IL‐1β, and IL‐6 from the Normal and CAD group recipients detected by quantitative ELISA‐based chemiluminescent assays (Biological replicates, n = 5). (E) Representative images of PAS and Masson staining in transplanted kidney tissues of recipients from the Normal and CAD groups (Biological replicates, n = 5. Scale bar = 20 µm). (F) Representative IHC staining images of FN, CD31 and TNFα expression in transplanted kidney tissues of recipients from the Normal and CAD groups (Biological replicates, n = 5. Scale bar = 20 µm). (G) The microvessel densities presented by IF staining assays for CD31 in transplanted kidney tissues of recipients from the Normal and CAD groups (Biological replicates, n = 5. Scale bar = 25 µm). (H) Results of quantitative ELISA‐based chemiluminescent assays for serum TNFα levels from Syn and Allo mice (Biological replicates, n = 5). (I) Representative images of PAS and Masson staining in renal allograft tissues of mice from the Syn and Allo groups (Biological replicates, n = 5. Scale bar = 20 µm). (J) Representative IF double staining images for colocalization of CD31 and αSMA in transplanted kidney tissues of mice from the Syn and Allo groups (Biological replicates, n = 3. Scale bar = 20 µm). (K) The microvessel densities presented by IHC staining for CD31 in transplanted kidney tissues of mice from the Syn and Allo groups (Biological replicates, n = 5. Scale bar = 20 µm). (L) The correlation analyses between TNFα and FN (R^2^ = 0.9921, *p* < 0.001), TNFα and CD31 (R^2^ = 0.9378, *p* < 0.001) in transplanted kidney tissues of mice from the Syn and Allo groups. (M,N) WB results and semi‐quantitative analyses of EMCN, CD31, Collagen I, αSMA and TNFα expression in transplanted kidney tissues of mice from the Syn and Allo groups (Biological replicates, n = 3). Statistical significance was assessed by two‐tailed Student's *t*‐test (D, G, H, K, and M, N) and Pearson correlation analysis and simple linear regression (L). Data were presented as mean ± SEM and *p < 0.05; **p < 0.01; ***p < 0.001; ns, not significant compared with Normal or Syn group.

### 2.2 Mitochondrial Dysfunction and Mitophagy Were Induced in Renal Vascular Endothelial Cells During the Progression of Renal Allograft Interstitial Fibrosis

Further analysis of the GSE195718 dataset revealed enhanced oxidative stress and activation of autophagy in the CAD group (Figure 1C). Considering the established association between oxidative stress and mitochondrial impairment, we next investigated mitochondrial dysfunction in transplant kidneys. Transmission electron microscopy (TEM) analyses of renal endothelial cells from Allo mice demonstrated an accumulation of damaged mitochondria characterized by matrix swelling and rupture of the outer membranes compared with Syn mice (Figure 2A). Consistently, Dihydroethidium (DHE) staining revealed markedly increased ROS production in allograft tissues from the Allo group compared with the Syn group (Figure 2B). To further evaluate mitochondrial injury, we examined cytosolic cytochrome C (Cyto C) release, a canonical marker of mitochondrial outer membrane permeabilization [19]. IHC staining results showed elevated Cyto C and Cleaved Caspase 3 in endothelial cells of Allo grafts (Figure 2C), these findings were also corroborated by WB analyses (Figure 2D). Collectively, these results indicate that mitochondrial dysfunction is a prominent feature of vascular endothelial cells during the process of EndMT and interstitial fibrosis in renal allografts.

**FIGURE 2:**
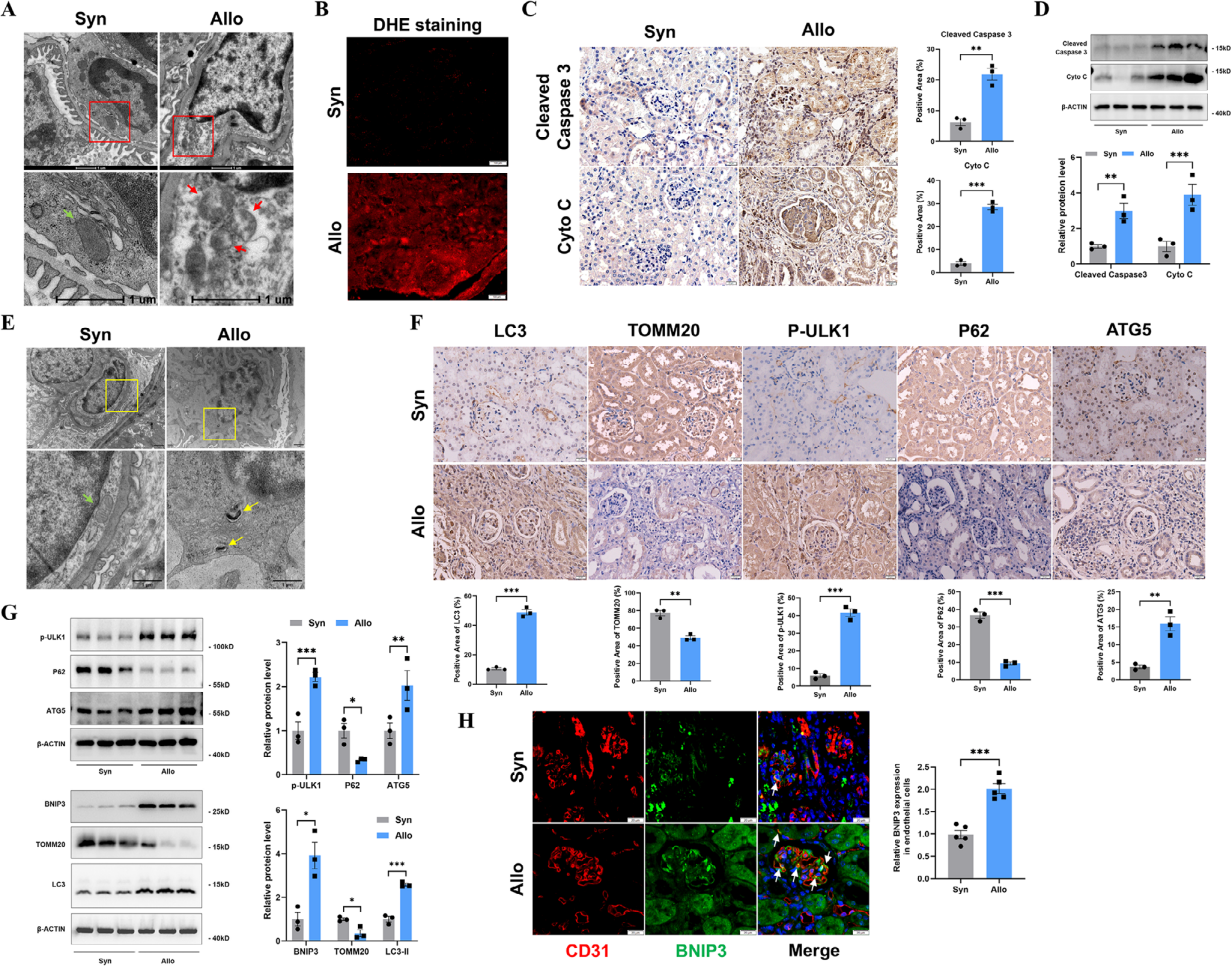
Mitochondrial dysfunction and mitophagy were induced in renal vascular endothelial cells during the progression of renal allograft interstitial fibrosis. (A) Representative TEM images of mitochondria in endothelial cells from transplanted kidneys of Syn and Allo mice (Scale bar = 1 µm). (Green arrowheads: normal mitochondria; Red arrowhead: damaged mitochondria). (B) Representative images of DHE staining in transplanted kidney tissues from Syn and Allo mice (Scale bar = 100 µm). (C) Representative IHC staining images and semi‐quantitative statistical analyses of Cleaved Caspase 3 and Cyto C expression and distribution in transplanted kidney tissues of mice from the Syn and Allo groups (Biological replicates, n = 3. Scale bar = 20 µm). (D) WB results and semi‐quantitative analyses of Cleaved Caspase 3 and Cyto C expression in transplanted kidney tissues of mice from the Syn and Allo groups (Biological replicates, n = 3). (E) Representative TEM images of mitophagosomes in endothelial cells from transplanted kidneys of Syn and Allo mice (Scale bar = 1 µm) (Yellow arrowhead: mitophagosome). (F) Representative IHC staining images and semi‐quantitative statistical analyses of LC3, TOMM20, p‐ULK1, P62, and ATG5 expression and distribution in transplanted kidney tissues of mice from the Syn and Allo groups (Biological replicates, n = 3. Scale bar = 20 µm). (G) WB results and semi‐quantitative analyses of p‐ULK1, P62, ATG5, LC3, TOMM20, and BNIP3 expression in transplanted kidney tissues of mice from the Syn and Allo groups (Biological replicates, n = 3). (H) Representative IF double staining images for colocalization of CD31 and BNIP3 (White arrows) in transplanted kidney tissues of mice from the Syn and Allo groups (Biological replicates, n = 5. Scale bar = 20 µm). Statistical significance was assessed by two‐tailed Student's *t*‐test (C,D and F–H). Data were presented as mean ± SEM and *p < 0.05; **p < 0.01; ***p < 0.001; ns, not significant compared with the Syn group.

Given the essential role of mitophagy in maintaining mitochondrial quality by eliminating dysfunctional organelles [16], we next examined whether its activity differed between Syn and Allo mice. TEM analyses revealed a significant increase in mitophagosomes in endothelial cells from the Allo group compared with Syn controls (Figure 2E). Consistently, WB analyses demonstrated activation of BNIP3‐dependent mitophagy in the Allo group, as evidenced by decreased TOMM20 and P62 levels accompanied by elevated p‐ULK1, ATG5, LC3‐II, and BNIP3 expression (Figure 2G). IHC staining produced concordant results (Figure 2F), while immunofluorescence (IF) double staining assays further confirmed increased BNIP3 specifically in endothelial cells (Figure 2H). Together, these findings indicate that mitochondrial impairment is accompanied by compensatory activation of mitophagy in vascular endothelial cells during the progression of renal allograft interstitial fibrosis. This prompted us to investigate whether inflammatory cues, particularly TNFα, contribute to the regulation of BNIP3‐mediated mitophagy in this context.

### 2.3 TNFα Neutralization With infliximab Ameliorated Renal Allograft Interstitial Fibrosis

To further investigate the role of TNFα in renal allograft interstitial fibrosis, we administered infliximab—an effective TNFα antagonist—to murine models of renal allogeneic transplantation. Serum TNFα levels were significantly elevated in saline‐treated Allo mice compared with Syn controls, whereas infliximab treatment reversed this increase (Figure 3A). Infliximab also improved renal allograft function, as reflected by reduced serum creatinine (Cr) and blood urea nitrogen (BUN) levels in Allo mice treated with infliximab, compared with the Allo group (Figure 3B). Histological analyses revealed attenuated tubular injury, glomerulosclerosis, and collagen deposition in allograft tissues from infliximab‐treated Allo mice (Figure 3C). Consistent with these findings, WB and IHC staining showed increased EMCN and CD31, along with decreased FN expression in infliximab‐ versus saline‐treated Allo mice, indicating that infliximab treatment could reduce EndMT (Figure 3D–F). In addition, microvessel densities were significantly increased in infliximab‐treated Allo group compared with the Allo group (Figure 3F). Furthermore, infliximab treatment diminished Cyto C release in the Allo group (Figure 3G), suggesting improvement of mitochondrial integrity. TEM analyses confirmed a marked reduction in damaged mitochondria following infliximab administration (Figure 3H). In addition, infliximab treatment was associated with reduced BNIP3‐mediated mitophagy markers in the Allo group (Figure 3I,J). Collectively, these findings show that TNFα is associated with mitochondrial injury, EndMT and renal allograft interstitial fibrosis, and is accompanied by induction of BNIP3‐associated mitophagy markers; conversely, TNFα neutralization improves renal allograft pathology while reducing these mitophagy signals in renal allografts.

**FIGURE 3:**
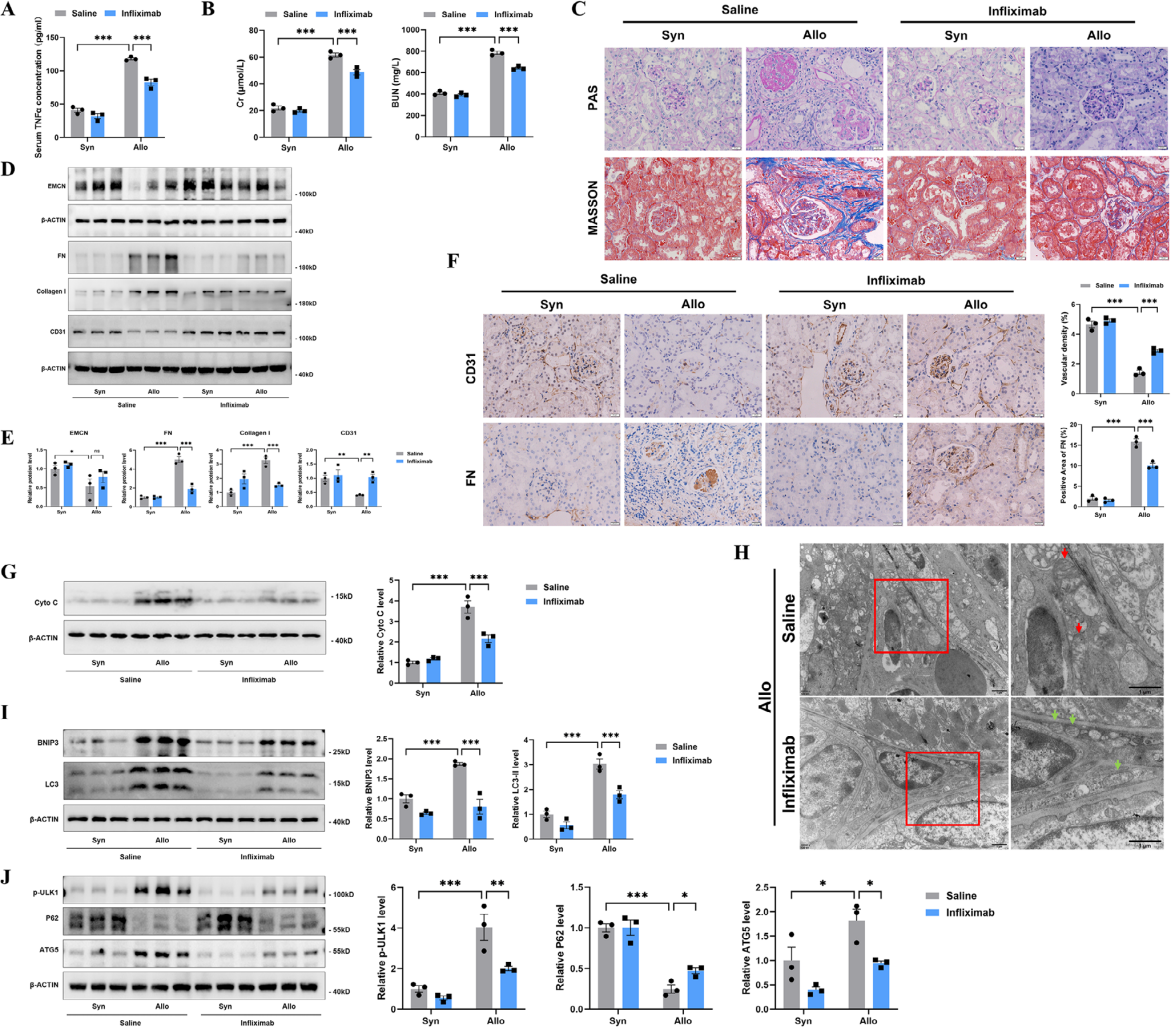
Infliximab treatment alleviated renal allograft interstitial fibrosis. Mice from the Syn and Allo groups were administered intraperitoneal injections of either saline (control) or infliximab every 4 weeks following kidney transplantation. Transplanted kidney tissues from different groups were subsequently collected at 16 weeks after kidney transplantation for further analyses. (A) Quantitative ELISA‐based chemiluminescent measurements of TNFα levels in serum from mice from different groups (Biological replicates, n = 3). (B) Serum Cr and BUN levels of mice from different groups (Biological replicates, n = 3). (C) Representative images of PAS and Masson staining in transplanted kidney tissues of mice from different groups (Biological replicates, Scale bar = 20 µm). (D,E) WB results (D) and semi‐quantitative analyses (E) of EMCN, FN, Collagen I and CD31 expression in transplanted kidney tissues of mice from different groups (Biological replicates, n = 3). (F) The microvessel densities for CD31 and the area of FN presented by IHC staining in transplanted kidney tissues of mice from different groups (Biological replicates, n = 3. Scale bar = 20 µm). (G) WB results and semi‐quantitative analyses of Cyto C expression in transplanted kidney tissues of mice from different groups (Biological replicates, n = 3). (H) Representative TEM images of mitochondria in endothelial cells from transplanted kidneys of mice from different groups (Scale bar = 1 µm). (Green arrowheads: normal mitochondria; Red arrowhead: damaged mitochondria). (I,J) WB results and semi‐quantitative analyses of BNIP3, LC3, p‐ULK1, P62, and ATG5 expression in transplanted kidney tissues of mice from different groups (Biological replicates, n = 3). Statistical significance was assessed by two‐way ANOVA with Sidak's post hoc test (A, B, E–G, and I, J). Data were presented as mean ± SEM and *p < 0.05; **p < 0.01; ***p < 0.001; ns, not significant compared with Saline+Syn or Saline+Allo group.

### 2.4 TNFα Induced Mitochondrial Dysfunction and Enhanced BNIP3‐Mediated Mitophagy In Vitro

To further elucidate the role of TNFα in EndMT and its underlying mechanism, we treated HUVECs with recombinant TNFα protein. As anticipated, TNFα induced EndMT in a dose‐dependent manner (Figures 4A,B and S2A,B). Transcriptomic profiling revealed that TNFα‐upregulated DEGs were significantly enriched in biological processes including Cyto C release from mitochondria and positive regulation of mitochondrial membrane permeability during apoptosis, as identified by GSEA (Figure 4C). WB analyses confirmed a dose‐dependent increase in Cyto C expression (Figures 4D and S2C). TEM analyses demonstrated the accumulation of structurally impaired mitochondria with ruptured outer membranes following TNFα treatment (Figures 4E and S2D). Morphological assessment using MitoTracker Red staining assays and ImageJ Mitochondria Analyzer plugin [27] indicated that TNFα promoted mitochondrial fragmentation, as reflected by reductions in mean area, perimeter, aspect ratio, and form factor (Figures 4F and S2E). In addition, TNFα stimulation decreased mitochondrial membrane potential and increased mitochondrial ROS production (Figures 4G,H and S2F). Collectively, these findings show that mitochondrial dysfunction is observed in parallel with TNFα‐induced EndMT in HUVECs.

**FIGURE 4:**
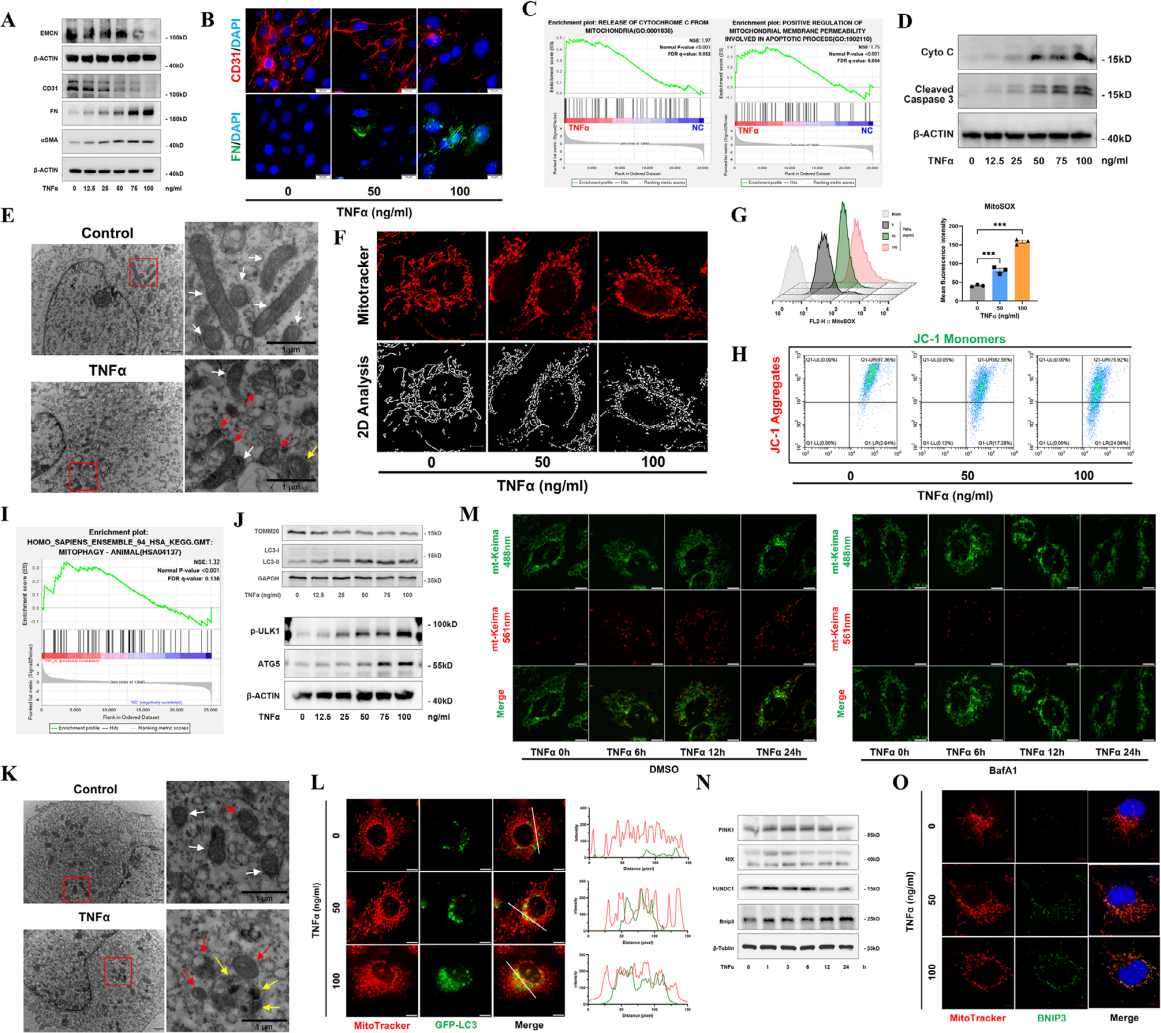
TNFα induced mitochondrial dysfunction and enhanced BNIP3‐mediated mitophagy in vitro. HUVECs were treated with 0–100 ng/mL TNFα for 24 h, or were treated with 100 ng/mL TNFα for 0–24 h, then cells were subsequently collected for further analyses. (A) WB results of EMCN, CD31, FN, and αSMA expression in HUVECs treated with different concentrations of TNFα (Biological replicates, n = 3). (B) Representative images of IF staining assays for CD31 and FN expression and distribution in HUVECs treated with different concentrations of TNFα (Biological replicates, n = 4. Scale bar = 20 µm). (C) GSEA enrichment analyses of differentially expressed genes identified by RNA‐seq in control and TNFα‐treated HUVECs. (D) WB results of Cyto C and Cleaved Caspase 3 expression in HUVECs treated with different concentrations of TNFα (Biological replicates, n = 3). (E) Representative TEM images of mitochondria in control and TNFα‐treated HUVECs (Biological replicates, n = 6. Scale bar = 1 µm). (White arrowheads: normal mitochondria; Red arrowhead: damaged mitochondria; Yellow arrowhead: autophagosome). (F) Representative images and quantitative analyses of MitoTracker Red staining assays in HUVECs treated with different concentrations of TNFα, analyzed using the Mitochondria Analyzer plugin for ImageJ (Biological replicates, n = 11. Scale bar = 5 µm). (G) Representative plots and quantitative analyses of flow cytometry assays for MitoSOX in HUVECs treated with different concentrations of TNFα (Biological replicates, n = 3). (H) Representative plots of flow cytometry assays for JC‐1 staining in HUVECs treated with different concentrations of TNFα (Biological replicates, n = 3). (I) GSEA enrichment analysis of differentially expressed genes identified by RNA‐seq in control and TNFα‐treated HUVECs. (J) WB results of TOMM20, LC3, p‐ULK1, and ATG5 expression in HUVECs treated with different concentrations of TNFα (Biological replicates, n = 3). (K) Representative TEM images of mitophagosomes in control and TNFα‐treated HUVECs (Biological replicates, n = 6. Scale bar = 1 µm). (White arrowheads: normal mitochondria; Red arrowhead: damaged mitochondria; Yellow arrowhead: mitophagosome). (L) Representative images and line‐profile analyses of IF staining assays for MitoTracker and GFP‐LC3 in HUVECs treated with different concentrations of TNFα (Biological replicates, n = 3. Scale bar = 10 µm). (M) Representative images of mt‐Keima‐based mitophagy signal assays in HUVECs treated with 100 ng/ml TNFα for different times and BafA1 (100 nM) for 4 h (Biological replicates, n = 8. Scale bar = 10 µm). (N) WB results of PINK1, NIX, FUNDC1, and BNIP3 expression in HUVECs treated with 100 ng/ml TNFα for different times (Biological replicates, n = 3). (O) Representative images of IF staining assays for MitoTracker and BNIP3 in HUVECs treated with different concentrations of TNFα (Biological replicates, n = 5. Scale bar = 5 µm). Statistical significance was assessed by one‐way ANOVA with Dunnett's post hoc test (G). Data were presented as mean ± SEM and ***p < 0.001 compared with control group.

Given that mitophagy is typically activated to eliminate dysfunctional mitochondria after mitochondrial damage [28], we investigated the effect of TNFα on mitophagy in HUVECs. GSEA revealed a significant enrichment of mitophagy‐related genes in response to TNFα treatment (Figure 4I). WB analyses showed increased p‐ULK1, ATG5, and LC3‐II, along with decreased TOMM20 expression, indicating enhanced mitophagic flux in HUVECs after TNFα treatment (Figures 4J and S2G). TEM analyses confirmed prominent mitophagosome formation (Figures 4K and S2H), which was supported by increased MitoTracker/LC3 colocalization (Figure 4L). Besides, the mt‐Keima–based mitophagy flux assays showed that TNFα markedly increased the lysosomal mt‐Keima signal, indicating enhanced mitochondrial delivery to acidic lysosomes (Figures 4M and S2I). Notably, co‐treatment with BafA1 significantly reduced the TNFα‐induced mt‐Keima red shift (Figures 4M and S2I). Accordingly, these results support the notion that TNFα enhances mitophagy flux, rather than reflecting a static accumulation due to impaired clearance. Since mitophagy is primarily activated through receptor‐mediated (e.g., BNIP3, FUNDC1, NIX) or PINK1/Parkin pathways [29], we next examined the key mediators. TNFα stimulation significantly upregulated BNIP3 expression (Figures 4N and S2J) and increased MitoTracker/BNIP3 colocalization (Figures 4O and S2K). Collectively, these results indicate that TNFα‐induced mitochondrial dysfunction could predominantly trigger BNIP3‐mediated mitophagy.

### 2.5 BNIP3 Knockdown Exacerbated Mitochondrial Dysfunction, EndMT and Renal Allograft Interstitial Fibrosis

The role of mitophagy in kidney fibrosis remains controversial. To examine the impact of BNIP3‐mediated mitophagy on renal allograft interstitial fibrosis, we administered adeno‐associated virus (AAV)‐shBNIP3 to mice following kidney transplantation. In the Allo group, BNIP3 knockdown significantly reduced mitophagy, as indicated by decreased BNIP3 and LC3 expression (Figure 5A). This reduction also diminished CD31 colocalization with LC3 and BNIP3 in renal endothelial cells, further confirming the suppression of BNIP3‐mediated mitophagy (Figure 5B). Furthermore, BNIP3 knockdown also promoted Cyto C release and aggravated mitochondrial damage in the endothelial cells (Figure 5C,D), indicating that the loss of BNIP3‐mediated mitophagy exacerbates mitochondrial dysfunction in the endothelial cells of renal allografts.

**FIGURE 5:**
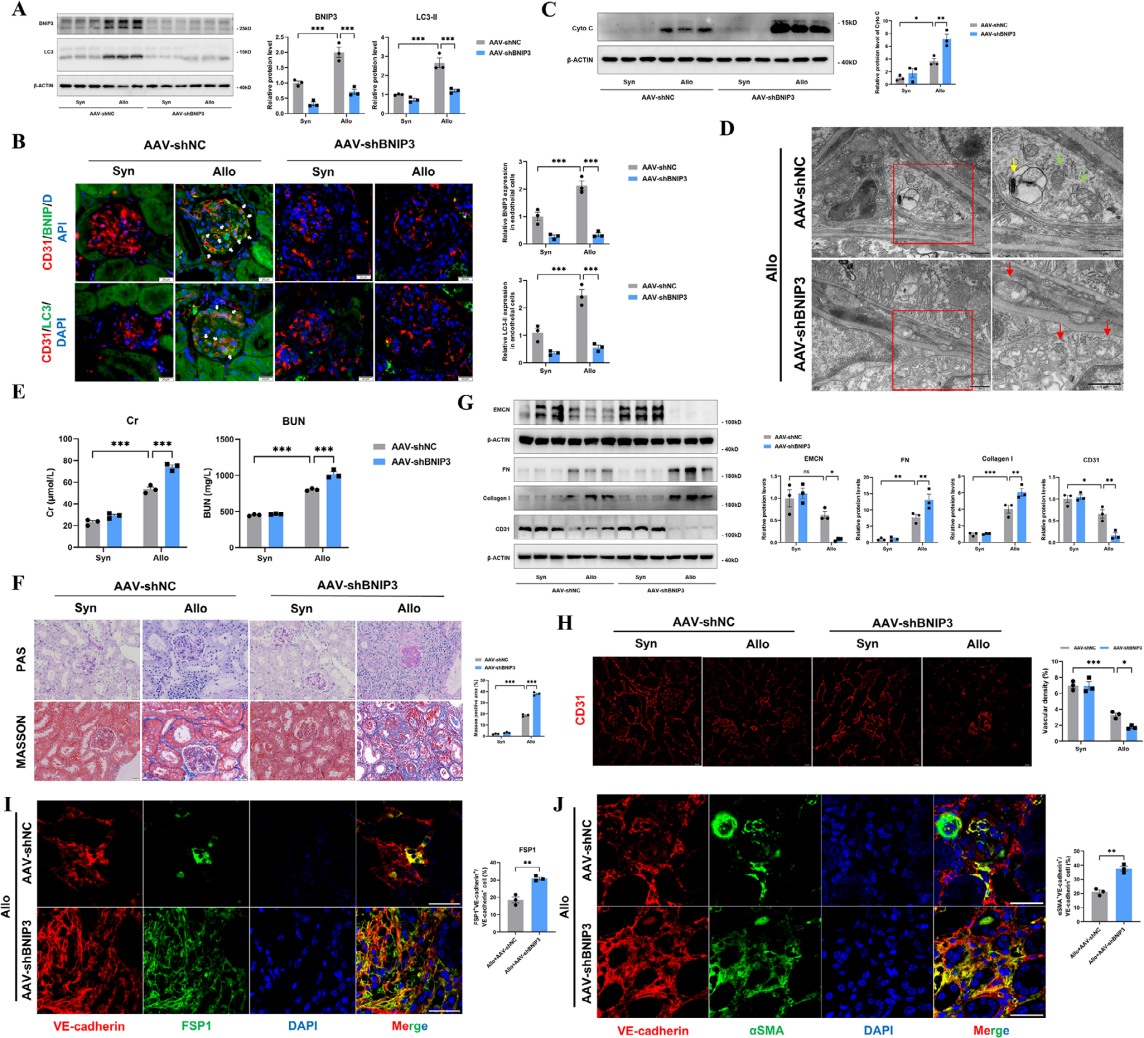
BNIP3 knockdown exacerbated mitochondrial dysfunction, EndMT and renal allograft interstitial fibrosis. Mice from the Syn and Allo groups received tail‐vein injections of AAV encoding BNIP3 shRNA (AAV‐shBNIP3) or a negative control AAV (AAV‐shNC) at 3 weeks after kidney transplantation. Transplanted kidney tissues were subsequently collected at 16 weeks after kidney transplantation for further analyses. (A) WB results and semi‐quantitative analyses of BNIP3 and LC3 expression in transplanted kidney tissues of mice from different groups (Biological replicates, n = 3). (B) Representative IF double‐staining images and quantitative analyses of CD31 colocalization with LC3 or BNIP3 (white arrows) in transplanted kidney tissues of mice from different groups (Biological replicates, n = 3. Scale bar = 20 µm). (C) WB results and semi‐quantitative analyses of Cyto C expression in transplanted kidney tissues of mice from different groups (Biological replicates, n = 3). (D) Representative TEM images of mitochondria in endothelial cells from transplanted kidneys of mice from different groups (Scale bar = 1 µm). (Green arrowheads: normal mitochondria; Red arrowhead: damaged mitochondria; Yellow arrowhead: mitophagosome). (E) Serum Cr and BUN levels of mice from different groups (Biological replicates, n = 3). (F) Representative images and semi‐quantitative analyses of PAS and Masson staining in transplanted kidney tissues of mice from different groups (Biological replicates, n = 3. Scale bar = 20 µm). (G) WB results and semi‐quantitative analyses of EMCN, FN, Collagen I and CD31 expression in transplanted kidney tissues of mice from different groups (Biological replicates, n = 3). (H) The microvessel densities presented by IF staining assays for CD31 in transplanted kidney tissues of mice from different groups (Biological replicates, n = 3. Scale bar = 25 µm). (I,J) Representative IF images co‐stained for VE‐cadherin and FSP1 or αSMA and quantification of VE‐cadherin^+^FSP1^+^ and VE‐cadherin^+^αSMA^+^ double‐positive cells in transplanted kidney tissues of mice from different groups (Biological replicates, n = 3. Scale bar = 20 µm). Statistical significance was assessed by two‐way ANOVA with Sidak's post hoc test (A–C, and E–J). Data were presented as mean ± SEM and *p < 0.05; **p < 0.01; ***p < 0.001; ns, not significant compared with Syn+AAV‐shNC or Allo+AAV‐shNC group.

We further observed that BNIP3 knockdown markedly increased serum Cr and BUN levels in the Allo group, indicating impairment of renal allograft function (Figure 5E). Histological analyses using PAS and Masson staining assays showed that BNIP3 knockdown aggravated tubular injury and interstitial fibrosis in the renal allografts (Figure 5F). Furthermore, BNIP3 knockdown enhanced EndMT and intensified allograft fibrosis (Figure 5G,I,J). In addition, microvessel densities were significantly decreased after BNIP3 knockdown (Figure 5H). Collectively, these findings highlight a protective role of BNIP3‐mediated mitophagy in mitigating renal allograft interstitial fibrosis.

### 2.6 BNIP3‐Mediated Mitophagy Mitigated TNFα‐Induced EndMT and Mitochondrial Dysfunction In Vitro

To investigate the role of BNIP3‐mediated mitophagy in TNFα‐induced EndMT and mitochondrial dysfunction, BNIP3 was silenced using small interfering RNA (siRNA). Knockdown of BNIP3 markedly attenuated TNFα‐induced mitophagy, evidenced by decreased p‐ULK1, ATG5, BNIP3, and LC3 expression and reduced MitoTracker/LC3 colocalization in HUVECs (Figure 6A,B). BNIP3 silencing further exacerbated TNFα‐induced mitochondrial dysfunction, including reduced mitochondrial membrane potential (Figure 6C), increased ROS generation (Figure 6D), and diminished mitochondrial area, perimeter, aspect ratio, and form factor (Figure 6E). To directly assess mitochondrial bioenergetic function, we conducted Seahorse Oxygen Consumption Rate (OCR) and Extracellular Acidification Rate (ECAR) assays. The results showed that TNFα markedly suppressed mitochondrial respiration, as evidenced by reduced basal respiration, maximal respiration, and ATP production, accompanied by a relative increase in ECAR (basal glycolysis and compensatory glycolysis), indicating a shift away from oxidative phosphorylation (Figure 6G,H). Notably, BNIP3 overexpression significantly restored basal respiration, maximal respiration, and ATP production, and attenuated the ECAR increase, supporting a BNIP3‐dependent rescue of mitochondrial bioenergetic capacity (Figure 6F–H). Besides, we estimated mitochondrial mass using MitoTracker Green staining assays. TNFα treatment tended to decrease the MitoTracker Green signal, whereas BNIP3 overexpression showed a tendency to increase the signal toward baseline (Figure 6I). Additionally, BNIP3 knockdown potentiated the TNFα‐induced EndMT phenotype (Figure 6J,K). In summary, these results indicate that BNIP3‐mediated mitophagy protects endothelial cells from TNFα‐induced mitochondrial damage and EndMT.

**FIGURE 6:**
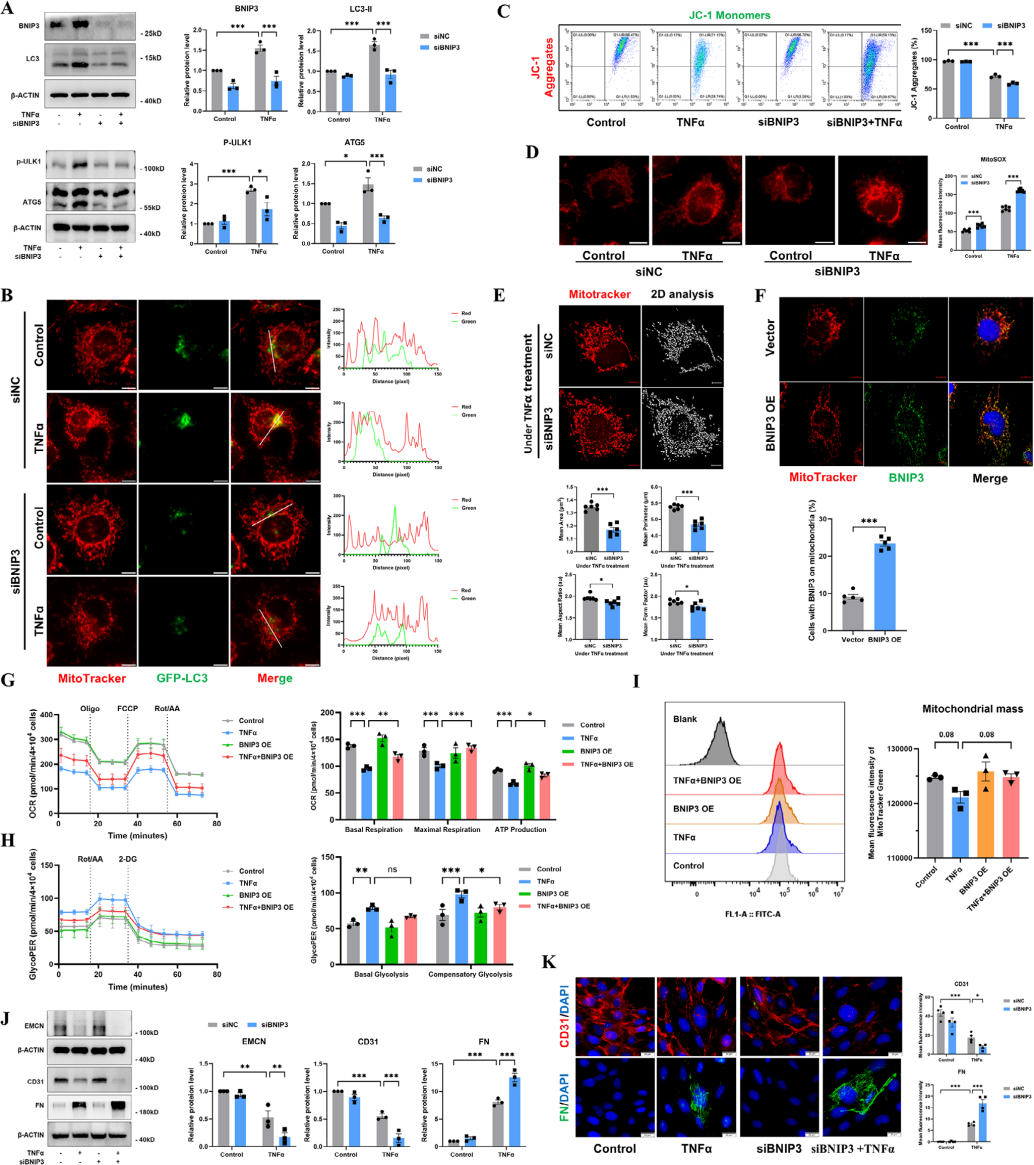
BNIP3‐mediated mitophagy mitigated TNFα‐induced EndMT and mitochondrial dysfunction in vitro. HUVECs were transfected with siNC or siBNIP3 for 24 h, followed by treatment with 100 ng/mL TNFα for an additional 24 h, then cells were subsequently collected for further analyses. (A) WB results and semi‐quantitative analyses of BNIP3, LC3, p‐ULK1, and ATG5 expression in HUVECs from different groups (Biological replicates, n = 3). (B) Representative images and line‐profile analyses of IF staining assays for MitoTracker and GFP‐LC3 in HUVECs from different groups (Biological replicates, n = 3. Scale bar = 10 µm). (C) Representative plots and quantitative analyses of flow cytometry assays for JC‐1 staining in HUVECs from different groups (Biological replicates, n = 3). (D) Representative images and quantitative analyses of IF staining assays for MitoSOX in HUVECs from different groups (Biological replicates, n = 3. Scale bar = 10 µm). (E) Representative images and quantitative analyses of MitoTracker Red staining assays in HUVECs from different groups, analyzed using the Mitochondria Analyzer plugin for ImageJ (Biological replicates, n = 6. Scale bar = 5 µm). HUVECs were transfected with vector or BNIP3 plasmid for 24 h, followed by treatment with 100 ng/mL TNFα for an additional 24 h, then cells were subsequently collected for further analyses. (F) Representative images and quantitative analyses of IF staining assays for MitoTracker and BNIP3 in HUVECs with or without BNIP3 overexpression (Biological replicates, n = 5. Scale bar = 5 µm). (G,H) Representative results of Seahorse OCR and ECAR assays in HUVECs from different groups (Biological replicates, n = 3). (I) Representative flow cytometry assay histograms and quantitative analyses of MitoTracker Green staining in HUVECs from different groups (Biological replicates, n = 3). (J) WB results and semi‐quantitative analyses of EMCN, CD31 and FN expression in HUVECs from different groups (Biological replicates, n = 3). (K) Representative images and quantitative analyses of IF staining assays for CD31 and FN expression and distribution in HUVECs from different groups (Biological replicates, n = 4. Scale bar = 20 µm). Statistical significance was assessed by two‐way ANOVA with Sidak's post hoc test (A, C, D, and G–K) and two‐tailed Student's *t*‐test (E and F). Data were presented as mean ± SEM and *p < 0.05; **p < 0.01; ***p < 0.001; ns, not significant compared with control/siNC+control, or TNFα/siNC+TNFα groups.

### 2.7 BNIP3 Transcription Upregulation Was Mediated by HIF‐1α

To elucidate the mechanism by which TNFα upregulates BNIP3 expression, proteasomal and autophagic degradation were inhibited using MG132 and chloroquine (CQ), respectively. Blocking these pathways did not significantly alter BNIP3 protein levels (Figure 7A), suggesting regulation may occur at the transcriptional level. Consistently, TNFα treatment increased BNIP3 mRNA in a dose‐dependent manner in HUVECs (Figure 7B). Besides, RNA stability assays revealed that TNFα did not significantly alter BNIP3 mRNA decay (Figure S3). KEGG‐based bioinformatics analysis identified several transcription factors potentially mediating BNIP3 transcription, including P65 (NF‐κB), E2F1, and HIF‐1α (Figure S4). Although we subsequently confirmed that TNFα activated NF‐κB signaling via P65 phosphorylation (Figure S5), P65 is known as a negative regulator of BNIP3 transcription [30]. In contrast, TNFα upregulated HIF‐1α expression without affecting E2F1 expression (Figure 7C), and IF staining confirmed increased HIF‐1α expression in TNFα‐treated HUVECs (Figure 7D). These findings support the hypothesis that HIF‐1α may be the transcription factor that mediates TNFα‐induced BNIP3 transcription.

**FIGURE 7:**
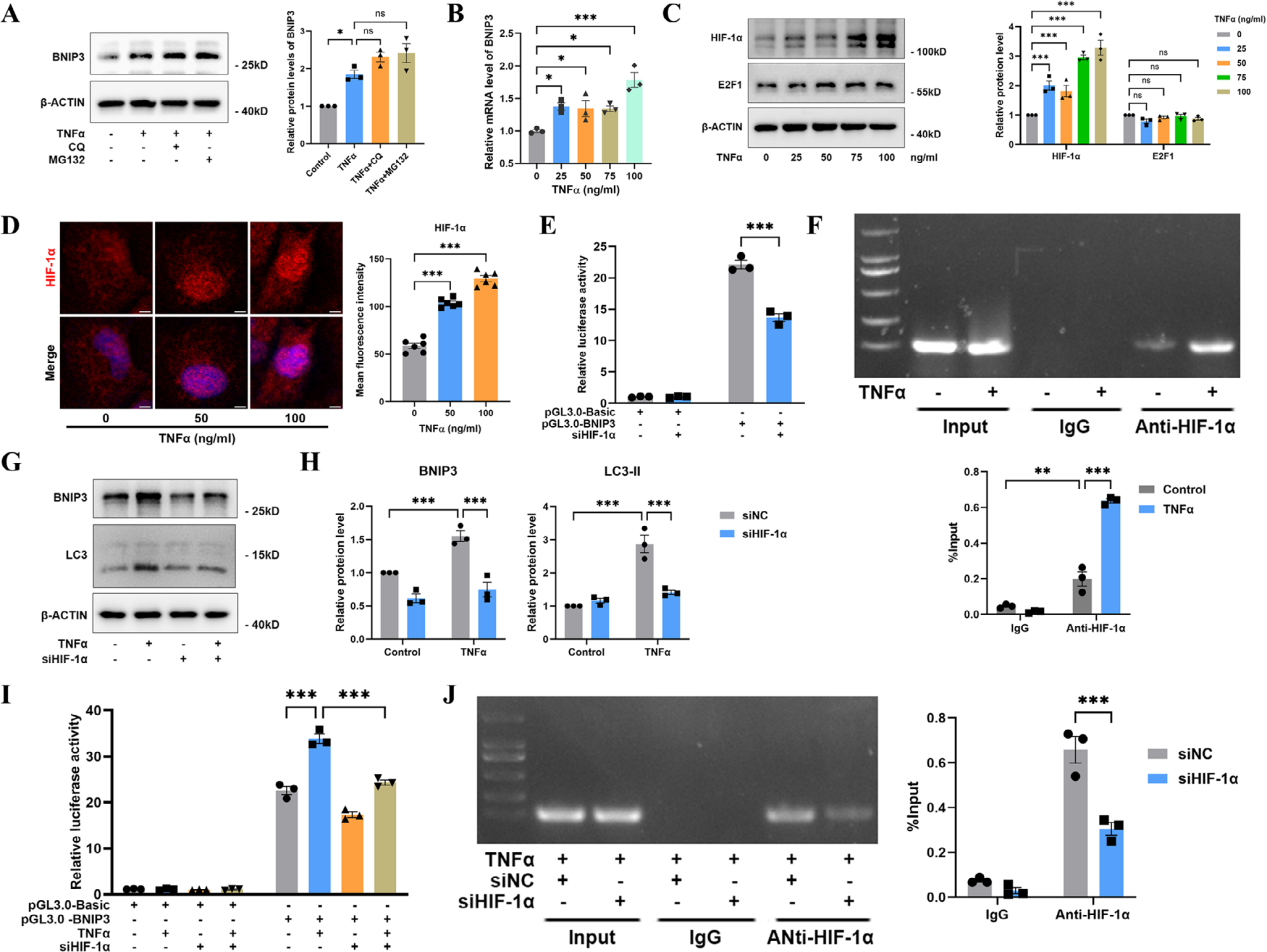
Upregulation of BNIP3 was mediated by HIF‐1α upregulation at the transcription level. (A) WB results and semi‐quantitative analyses of BNIP3 expression in HUVECs treated with 10 µM CQ for 24 h or 10 µM MG132 for 6 h, followed by 100 ng/ml TNFα stimulation for 24 h (Biological replicates, n = 3). (B) RT‐qPCR analyses of BNIP3 mRNA expression in HUVECs treated with different concentrations of TNFα for 24 h (Biological replicates, n = 3). (C) WB results and semi‐quantitative analyses of HIF‐1α and E2F1 expression in HUVECs treated with different concentrations of TNFα for 24 h (Biological replicates, n = 3). (D) Representative images and quantitative analyses of IF staining assays for HIF‐1α and nucleus in HUVECs treated with different concentrations of TNFα for 24 h (Biological replicates, n = 6. Scale bar = 5 µm). (E) Results of luciferase activity assays in HUVECs from different groups after knockdown of HIF‐1α or not. The pRL‐TK vector and the recombinant luciferase reporter pGL3.0‐BNIP3 were co‐transfected with siNC or siHIF‐1α into HUVECs. Cells were subsequently harvested to measure luciferase activity (Biological replicates, n = 3). **(F**) Representative results and semi‐quantitative analyses of ChIP assays for HIF‐1α in HUVECs treated with or without TNFα. HUVECs were treated with TNFα or control for 24 h, after which cells were harvested for ChIP assays. Chromatin fragments were immunoprecipitated using a HIF‐1α antibody, with IgG serving as the negative control (Biological replicates, n = 3). (G,H) WB results and semi‐quantitative analyses of BNIP3 and LC3 expression in HUVECs from different groups. HUVECs were transfected with siNC or siHIF‐1α for 24 h, followed by treatment with 100 ng/mL TNFα for an additional 24 h, then cells were harvested for WB assays. (Biological replicates, n = 3). (I) Results of luciferase activity assays in HUVECs from different groups after knockdown of HIF‐1α or not. HUVECs were transfected with siNC or siHIF‐1α for 24 h, followed by transfection with the pRL‐TK vector and the recombinant luciferase reporter pGL3.0‐BNIP3 for an additional 24 h. Afterward, cells were treated with control solvent or 100 ng/mL TNFα for 24 h and subsequently harvested to measure luciferase activity (Biological replicates, n = 3). (J) Representative results and semi‐quantitative analyses of ChIP assays for HIF‐1α in TNFα‐treated HUVECs after knockdown of HIF‐1α or not. HUVECs were transfected with siNC or siHIF‐1α for 24 h, followed by treatment with TNFα for an additional 24 h. Cells were then harvested for ChIP assays. Chromatin fragments were immunoprecipitated using a HIF‐1α antibody, with IgG serving as the negative control (Biological replicates, n = 3). Statistical significance was assessed by two‐way ANOVA with Sidak's post hoc test (A, E‐F, and H‐J) and one‐way ANOVA with Dunnett's post hoc test (B–D). Data were presented as mean ± SEM and *p < 0.05; **p < 0.01; ***p < 0.001; ns, not significant compared with control/siNC+control/pGL3.0‐BNIP3+siNC or TNFα/siNC+TNFα/pGL3.0‐BNIP3+siNC +TNFα groups.

To investigate potential mechanisms of HIF‐1α in regulating BNIP3, we used the JASPAR database to predict HIF‐1α binding sites in the BNIP3 promoter (relative score > 90%; Table S3) and constructed reporter plasmids. Dual‐luciferase reporter assays revealed that HIF‐1α knockdown reduced BNIP3 promoter activity (Figure 7E). Chromatin immunoprecipitation (ChIP)‐qPCR assays confirmed that TNFα enhanced direct binding of HIF‐1α to the BNIP3 promoter (Figure 7F). WB analyses demonstrated that HIF‐1α silencing markedly attenuated TNFα‐induced BNIP3‐mediated mitophagy (Figure 7G,H). Consistently, HIF‐1α siRNA abolished the TNFα‐induced increase in BNIP3 promoter activity and HIF‐1α binding to the BNIP3 promoter (Figure 7I) and HIF‐1α promoter binding (Figure 7J). Collectively, these results indicate that TNFα upregulates BNIP3 transcription by increasing HIF‐1α expression and promoter occupancy.

### 2.8 TNFα Inhibited Proteasome‐Dependent Degradation of HIF‐1α by Reducing K48‐Linked Ubiquitination at K532

To elucidate the mechanism by which TNFα regulates HIF‐1α expression, we initially assessed its transcriptional regulation. RT–qPCR assays showed that TNFα treatment did not alter HIF‐1α mRNA levels in HUVECs at any tested concentration (Figure 8A). We then evaluated protein stability using the protein synthesis inhibitor Cycloheximide (CHX). TNFα significantly prolonged HIF‐1α half‐life, indicating that it enhanced HIF‐1α protein stability (Figure 8B). Moreover, the proteasome inhibitor MG132, but not the autophagy inhibitor CQ, reversed HIF‐1α degradation (Figure 8C). Co‐immunoprecipitation (Co‐IP) assays revealed that TNFα reduced ubiquitination of endogenous HIF‐1α (Figure 8D), consistent with IF staining results (Figure 8E). These findings indicate that TNFα upregulates HIF‐1α by inhibiting ubiquitin‐proteasome–mediated protein degradation.

**FIGURE 8:**
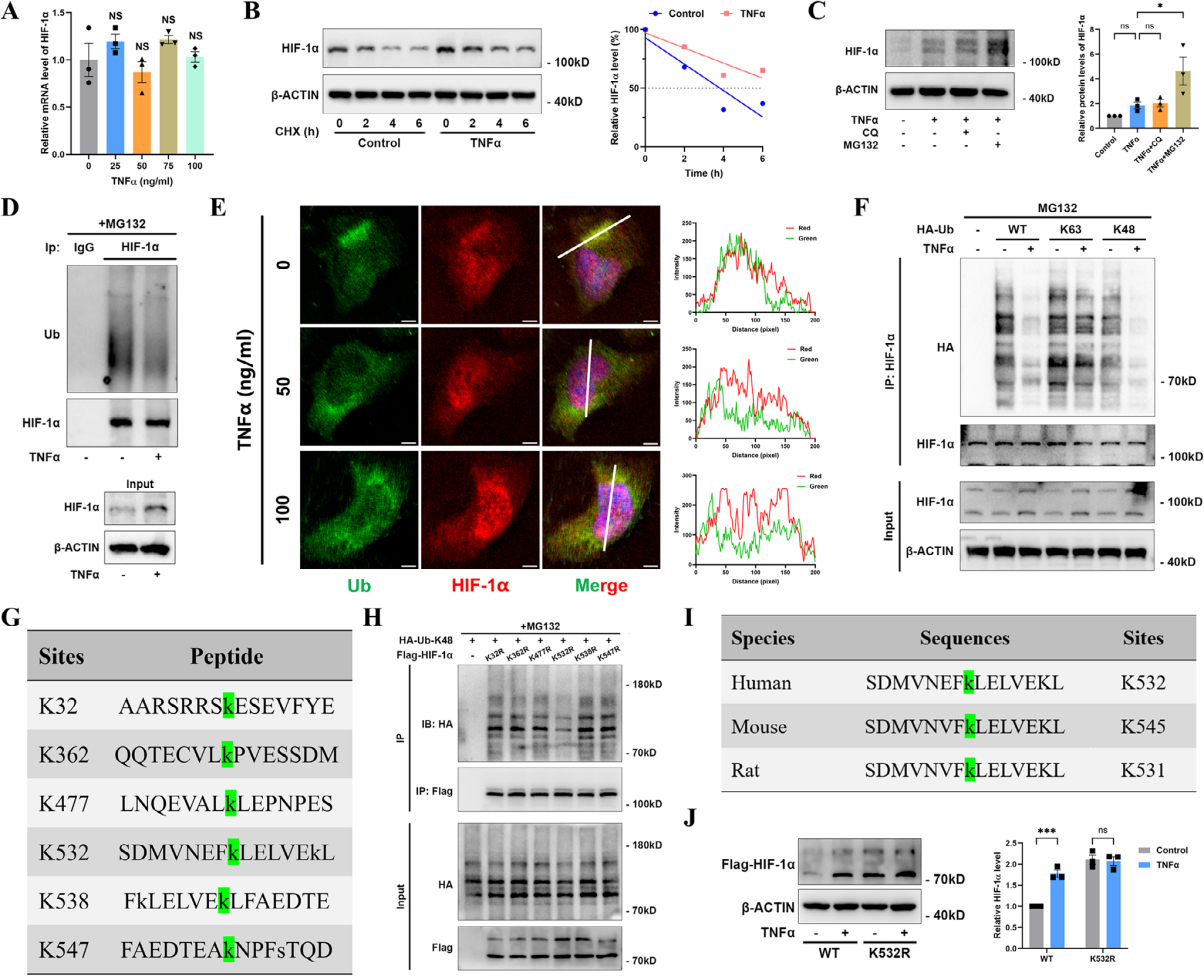
TNFα inhibited proteasome‐dependent degradation of HIF‐1α by reducing K48‐linked ubiquitination at K532. (A) Results of RT‐qPCR analyses of HIF‐1α mRNA expression in HUVECs treated with different concentrations of TNFα for 24 h (Biological replicates, n = 3). (B) WB results and semi‐quantitative analyses of CHX chase assay for HIF‐1α expression in HUVECs treated with CHX (200 µg/mL) and TNFα or CHX alone for the indicated time points (Biological replicates, n = 3). (C) WB results and semi‐quantitative analyses of HIF‐1α expression in HUVECs treated with TNFα and CQ/MG132 or TNFα alone. HUVECs treated with 10 µM CQ for 24 h or 10 µM MG132 for 6 h, followed by TNFα stimulation (Biological replicates, n = 3). (D) Results of co‐immunoprecipitation assays for ubiquitination of HIF‐1α in HUVECs treated with or without TNFα (Biological replicates, n = 3). (E) Representative images and line‐profile analyses of IF staining assays for Ubiquitin and HIF‐1α in HUVECs treated with different concentrations of TNFα for 24 h (Biological replicates, n = 3. Scale bar = 5 µm). (F) Results of co‐immunoprecipitation assays for HIF‐1α ubiquitination in HUVECs treated with or without TNFα following 48‐h transfection with WT‐Ub, K63‐linked Ub, or K48‐linked Ub plasmids (Biological replicates, n = 3). (G) Reported ubiquitination sites of human HIF‐1α according to the PhosphoSitePlus database (https://www.phosphosite.org/). (H) Results of co‐immunoprecipitation assays for HIF‐1α ubiquitination in HUVECs co‐transfected with HA‐Ub‐K48 and various HIF‐1α mutant plasmids (Biological replicates, n = 3). (I) The K532 residue of HIF‐1α is highly conserved among humans, mice, and rats. (J) WB results and semi‐quantitative analyses of Flag‐HIF‐1α expression in control or TNFα‐treated HUVECs transfected with either WT HIF‐1α or K532R HIF‐1α plasmids (Biological replicates, n = 3). Data were presented as mean ± SEM. *p < 0.05, **p < 0.01, ***p < 0.001. Statistical significance was assessed by one‐way ANOVA with Dunnett's post hoc test (A) and two‐way ANOVA with Sidak's post hoc test (C and J). Data were presented as mean ± SEM and *p < 0.05; **p < 0.01; ***p < 0.001; ns, not significant compared with control, WT HIF‐1α+control or K532R HIF‐1α+control between groups.

Mammalian cells employ diverse ubiquitin chain linkages, each mediating distinct biological functions. K48‐linked polyubiquitination primarily directs substrate proteins for proteasomal degradation, whereas K63‐linked chains typically activate specific signaling pathways. To identify the polyubiquitination pattern regulating HIF‐1α, we transfected HUVECs with plasmids encoding exclusively K48‐linked or K63‐linked ubiquitin chains. TNFα specifically reduced K48‐linked polyubiquitination of HIF‐1α without affecting K63‐linked chains (Figure 8F), indicating that TNFα regulates HIF‐1α protein stability via modulation of K48‐linked ubiquitination.

Subsequently, we sought to identify the lysine residue on HIF‐1α responsible for its ubiquitination. Based on the PhosphoSitePlus database, six lysine residues on human HIF‐1α have been reported as ubiquitination sites [31, 32, 33, 34, 35] (Figure 8G). To validate this, we generated Flag‐tagged HIF‐1α mutants in which individual lysines were substituted with arginine (K‐to‐R). Co‐IP assays revealed that the K532R mutation markedly reduced HIF‐1α ubiquitination, indicating that K532 is the critical residue for TNFα‐mediated HIF‐1α ubiquitination (Figure 8H). This residue is highly conserved across humans, mice, and rats (Figure 8I). Consistently, the K532R mutant exhibited significantly attenuated degradation in response to TNFα treatment (Figure 8J). Collectively, these results demonstrate that TNFα inhibits proteasome‐dependent degradation of HIF‐1α protein by specifically reducing its K48‐linked ubiquitination at the K532 residue.

### 2.9 NEDD4 Mediated the K48‐Linked Polyubiquitination of HIF‐1α at the K532 Residue, Thereby Promoting Its Proteasome‐Dependent Degradation

To identify the E3 ubiquitin ligase responsible for HIF‐1α protein ubiquitination and degradation, we immunoprecipitated HIF‐1α and analyzed the co‐precipitated proteins by liquid chromatography‐mass spectrometry (LC‐MS). This screen identified NEDD4 as the most enriched E3 ligase (Figure 9A). We validated this interaction via Co‐IP assays, confirming that NEDD4 was specifically precipitated by HIF‐1α antibodies (Figure 9B,C). This interaction was further verified using epitope‐tagged proteins in HUVECs (Figure 9D) and by confocal microscopy (Figure 9E). NEDD4 overexpression significantly accelerated HIF‐1α degradation, as shown by reduced protein half‐life in CHX chase assays (Figure 9F). To map the interacting domain, we expressed HA‐tagged NEDD4 fragments in HUVECs and found that deletion of the WW domain completely abolished the NEDD4‐HIF‐1α interaction, indicating that this domain is essential for binding (Figure 9G–I). Ubiquitination assays revealed that NEDD4 overexpression enhanced K48‐linked, but not K63‐linked, polyubiquitination of HIF‐1α (Figure 9J). Co‐transfection of HA‐NEDD4 and Flag‐HIF‐1α Wild‐type (WT) or K532R mutant plasmids showed significantly reduced ubiquitination in the Flag‐HIF‐1α K532R mutant plasmid compared to WT plasmid, identifying Lys532 as the primary site for NEDD4‐mediated ubiquitination (Figure 9K).

**FIGURE 9:**
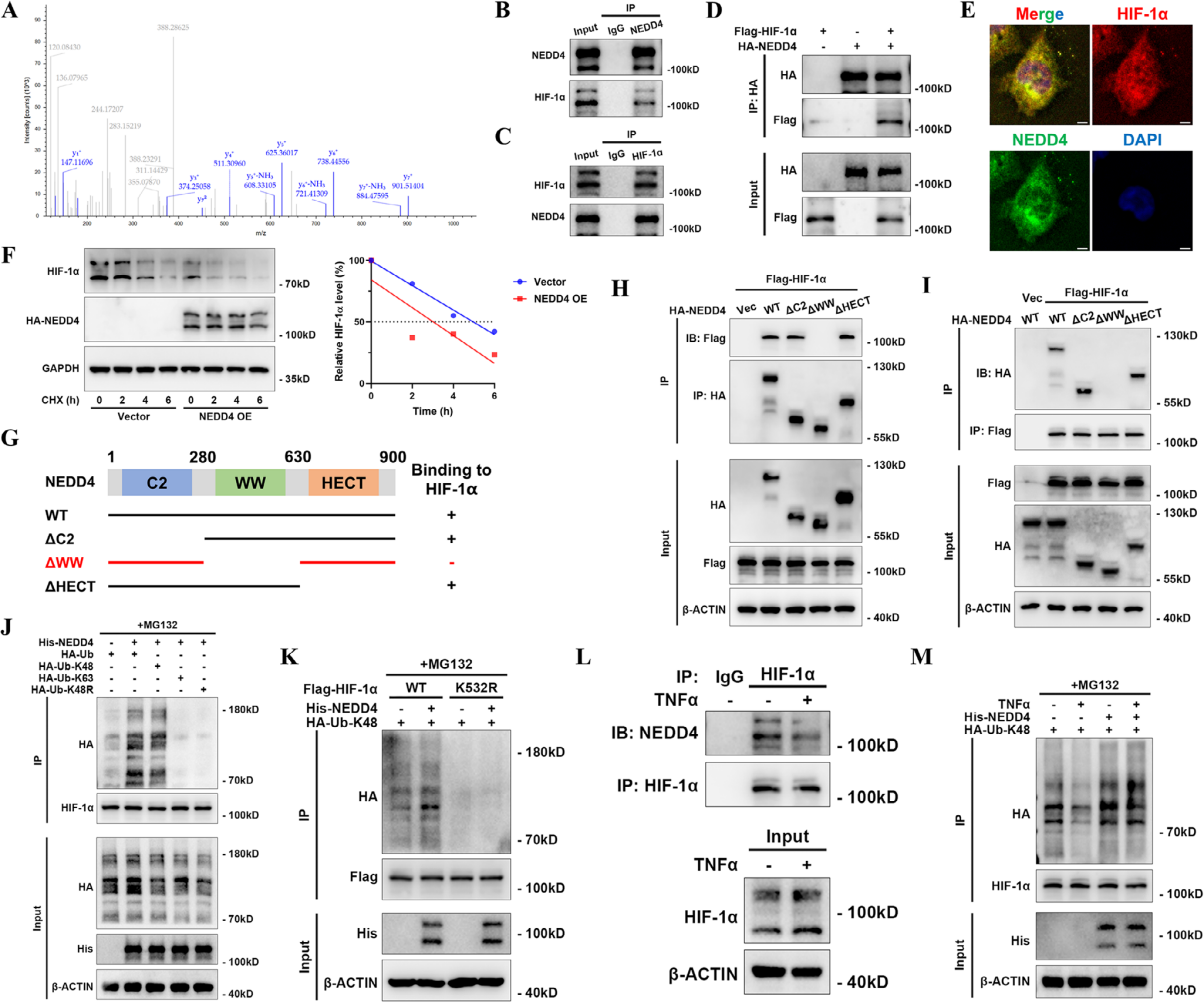
NEDD4 mediated the K48‐linked polyubiquitination of HIF‐1α at the K532 residue, thereby promoting its proteasomal degradation. (A) The mass spectrometry spectrum for NEDD4. (B,C) Results of co‐immunoprecipitation assays for interaction between NEDD4 and HIF‐1α in HUVECs (Biological replicates, n = 3). (D) Results of co‐immunoprecipitation assays for interaction between HA‐NEDD4 and Flag‐HIF‐1α in HUVECs (Biological replicates, n = 3). (E) Representative images of IF staining assays for HIF‐1α and NEDD4 expression and distribution in HUVECs (Biological replicates, n = 3. Scale bar = 5 µm). (F) WB results and semi‐quantitative analyses of CHX chase assays for HIF‐1α and NEDD4 expression in HUVECs transfected with either vector or HA‐NEDD4 plasmid and treated with CHX (200 µg/mL) for the indicated time points (Biological replicates, n = 3). (G) Schematic representation of WT NEDD4 and its deletion mutants, along with a summary of their interactions with HIF‐1α. (+: strong binding; −: no binding). (H,I) Results of co‐immunoprecipitation assays for binding sites of NEDD4 involved in interaction between NEDD4 and HIF‐1α in HUVECs. Flag‐HIF‐1α was co‐transfected with different HA‐NEDD4 deletion mutants or vector into HUVECs for 48 h, then cells were harvested for co‐immunoprecipitation assays (Biological replicates, n = 3). (J) Co‐immunoprecipitation results identifying the ubiquitin linkage site associated with HIF‐1α ubiquitination in HUVECs. HUVECs were treated with 10 µM MG132 for 6 h, and co‐transfected with His‐NEDD4 and HA‐Ub or various mutant plasmids for 48 h, then cells were harvested for co‐immunoprecipitation assay to assess the ubiquitination of HIF‐1α using the specified antibodies (Biological replicates, n = 3). (K) Results of co‐immunoprecipitation assays for ubiquitination site of HIF‐1α in HUVECs. HUVECs were treated with 10 µM MG132 for 6 h, and co‐transfected with HA‐Ub‐K48 and WT‐Flag‐HIF‐1α±His‐NEDD4 plasmids or K532R‐Flag‐HIF‐1α±His‐NEDD4 plasmids for 48 h, then co‐immunoprecipitation assay was performed to evaluate the ubiquitination of Flag‐HIF‐1α in HUVECs. (Biological replicates, n = 3). (L) Results of co‐immunoprecipitation assays for interaction between NEDD4 and HIF‐1α in HUVECs treated with TNFα. HUVECs were treated with or without TNFα for 24 h, then a co‐immunoprecipitation assay was performed to examine the interaction between NEDD4 and HIF‐1α (Biological replicates, n = 3). (M) Results of co‐immunoprecipitation assays for ubiquitin linkage type involved in HIF‐1α ubiquitination regulated by TNFα in HUVECs. HUVECs were treated with 10 µM MG132 for 6 h, and co‐transfected with HA‐Ub‐K48 and His‐NEDD4 plasmids or HA‐Ub‐K48 plasmid alone for 48 h, then cells were treated with or without TNFα. Cells were harvested for a co‐immunoprecipitation assay to examine the ubiquitination of HIF‐1α (Biological replicates, n = 3).

We next examined how TNFα regulates this proteasome‐dependent degradation pathway. Co‐IP assays showed that TNFα treatment weakened the interaction between NEDD4 and HIF‐1α (Figure 9L). Consistently, NEDD4 overexpression restored the TNFα‐induced reduction in K48‐linked ubiquitination of HIF‐1α (Figure 9M). Taken together, these results demonstrate that NEDD4 acts as a functional E3 ubiquitin ligase that promotes HIF‐1α K48‐linked ubiquitination at Lys532.

### 2.10 NEDD4 Was Crucial for TNFα‐Induced BNIP3‐Mediated Mitophagy

We next investigated the role of NEDD4 in TNFα‐induced mitophagy in endothelial cells. NEDD4 overexpression decreased HIF‐1α expression and its nuclear translocation induced by TNFα (Figure 10A). Moreover, NEDD4 overexpression significantly suppressed BNIP3 and LC3 expression and diminished MitoTracker/LC3 colocalization in HUVECs, thereby abrogating TNFα‐induced BNIP3‐mediated mitophagy (Figure 10B–D). Consistently, NEDD4 overexpression elevated mitochondrial ROS production in HUVECs treated with TNFα, reflecting impaired mitophagy (Figure 10E). In TNFα‐stimulated HUVECs, NEDD4 overexpression also exacerbated mitochondrial damage, as indicated by reductions in mitochondrial area, perimeter, aspect ratio, and form factor (Figure 10F). Collectively, these findings demonstrate that NEDD4 functions as a crucial negative regulator of TNFα‐induced mitophagy via the NEDD4‐HIF‐1α‐BNIP3 pathway.

**FIGURE 10:**
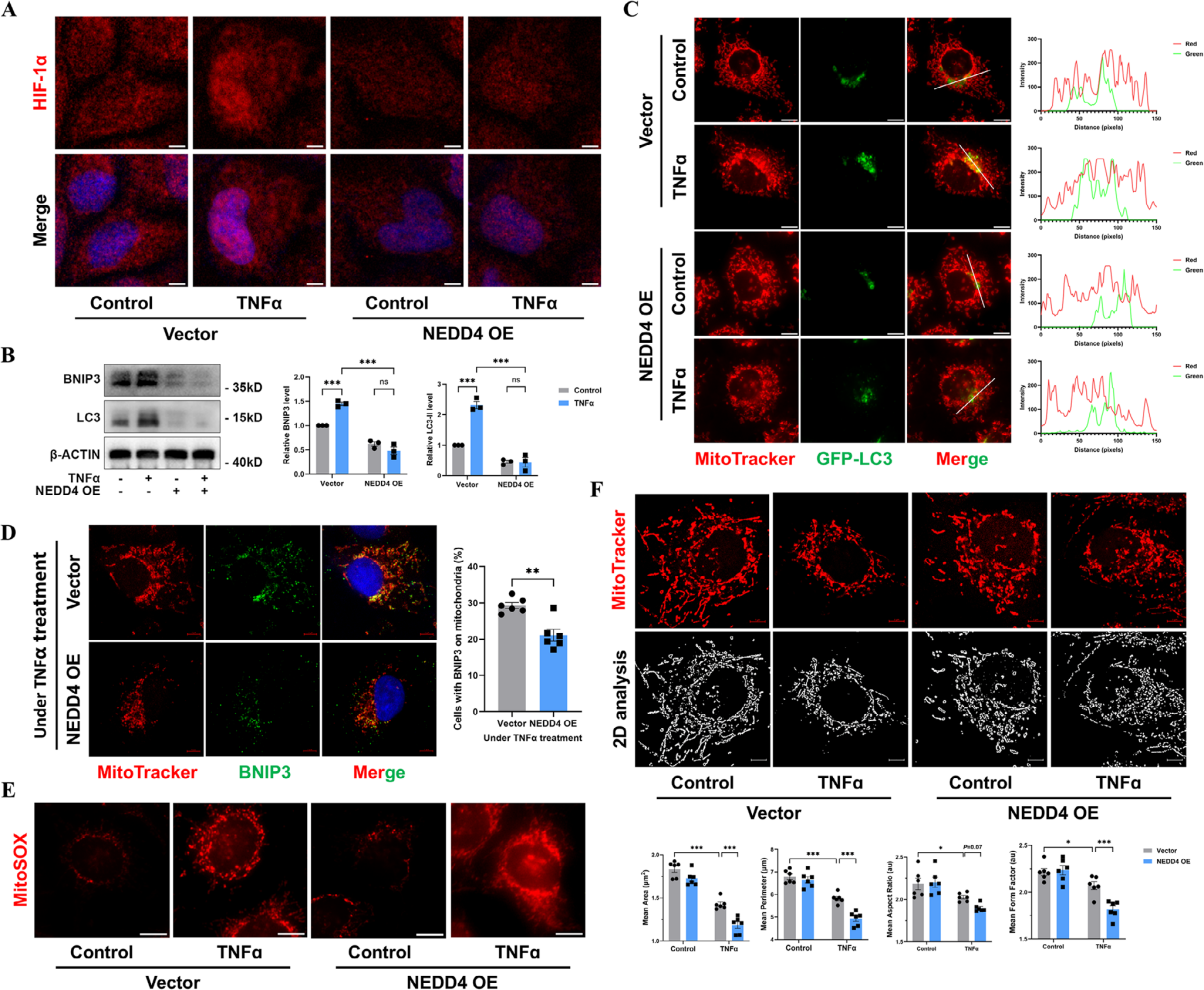
NEDD4 was crucial for TNFα‐induced BNIP3‐mediated mitophagy. HUVECs were transfected with vector or NEDD4 overexpression plasmids for 24 h, followed by treatment with 100 ng/mL TNFα for an additional 24 h or not. (A) Representative images and quantitative analyses of IF staining assays for HIF‐1α in HUVECs from different groups (Biological replicates, n = 3. Scale bar = 5 µm). (B) WB results and semi‐quantitative analyses of BNIP3 and LC3 expression in HUVECs from different groups (Biological replicates, n = 3). (C) Representative images and line‐profile analyses of IF staining assays for MitoTracker and GFP‐LC3 in HUVECs from different groups (Biological replicates, n = 3. Scale bar = 10 µm). (D) Representative IF double staining images and quantitative analyses for colocalization of MitoTracker and BNIP3 in HUVECs from different groups (Biological replicates, n = 6. Scale bar = 5 µm). (E) Representative images and quantitative analyses of IF staining assays for MitoSOX in HUVECs from different groups (Biological replicates, n = 3. Scale bar = 10 µm). (F) Representative images and quantitative analyses of MitoTracker Red staining assays in HUVECs from different groups, analyzed using the Mitochondria Analyzer plugin for ImageJ (Biological replicates, n = 6. Scale bar = 5 µm). Statistical significance was assessed by two‐way ANOVA with Sidak's post hoc test (B and F) and two‐tailed Student's *t*‐test (D). Data were presented as mean ± SEM and *p < 0.05; **p < 0.01; ***p < 0.001; ns, not significant compared with Vector+control, NEDD4 OE+control or Vector+TNFα groups.

### 2.11 Endothelial HIF‐1α Deletion Aggravated Renal Allograft Interstitial Fibrosis

To investigate the role of endothelial HIF‐1α expression in renal allograft interstitial fibrosis, we generated endothelial cell‐specific HIF‐1α knockout mice using the Cre‐LoxP system and established a chronic renal allograft fibrosis model (Figure 11B). Recipient BALB/c mice received kidneys from either *HIF‐1α^fl/fl^ Cre^+/−^* donors (HIF‐1α^−/−^ Allo group) or *HIF‐1α^fl/fl^ Cre^−/−^* donors (WT Allo group) (Figure 11A). WB and IF staining analyses confirmed a significant reduction of HIF‐1α expression in the HIF‐1α knockout allografts (Figure 11C,D).

**FIGURE 11:**
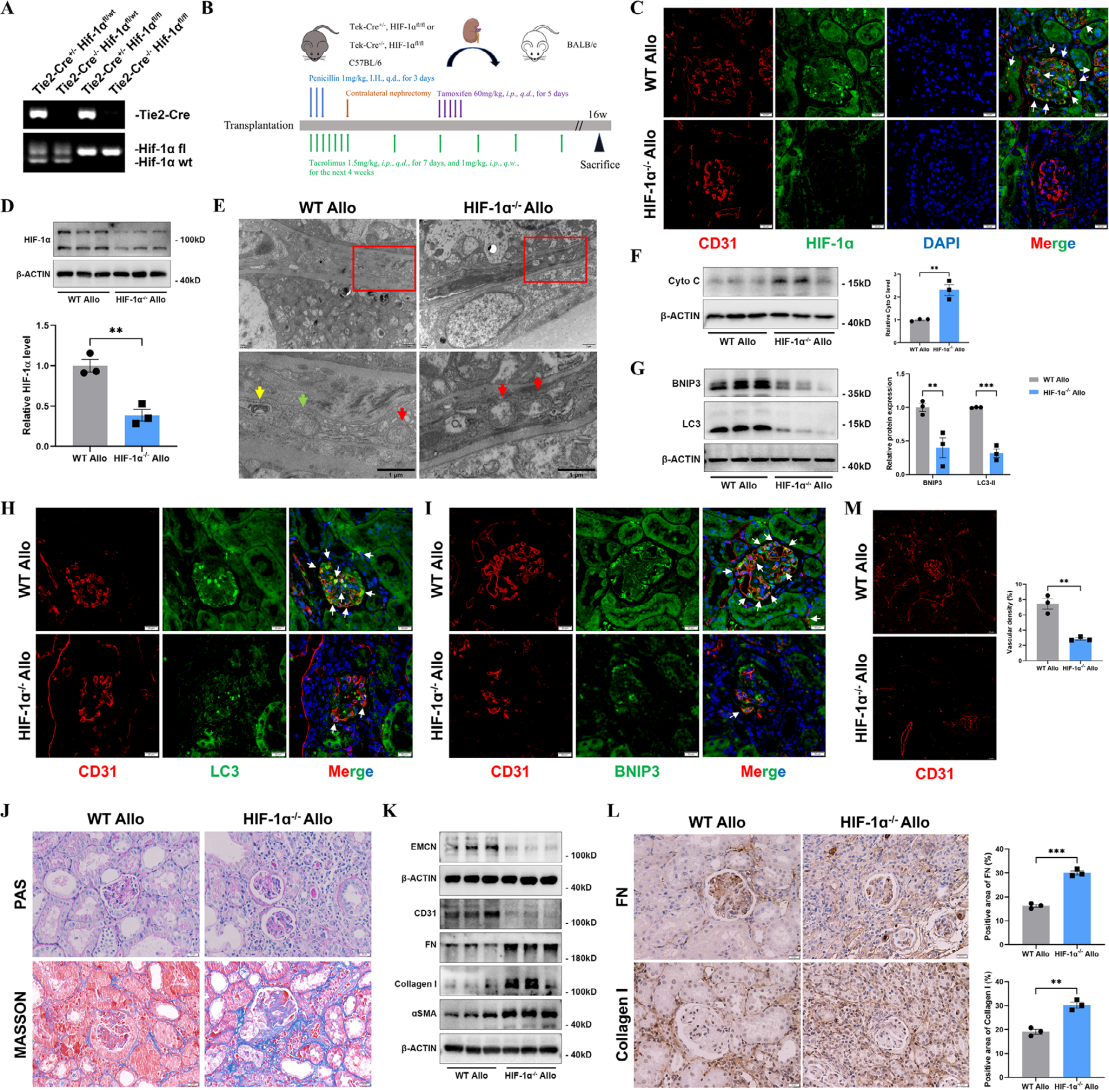
Endothelial HIF‐1α deletion aggravated renal allograft interstitial fibrosis. (A) Representative PCR results for genotyping mice using genomic DNA. (B) Schematic diagram of the surgical procedure for the murine renal allogeneic transplantation model in the WT Allo and HIF‐1α^−/−^ Allo groups. (C) Representative IF double‐staining images for colocalization of CD31 and HIF‐1α (white arrows) in transplanted kidney tissues of mice from the WT Allo and HIF‐1α^−/−^ Allo groups (Biological replicates, n = 3. Scale bar = 20 µm). (D) WB results and semi‐quantitative analyses of HIF‐1α expression in transplanted kidney tissues of mice from the WT Allo and HIF‐1α^−/−^ Allo groups (Biological replicates, n = 3). (E) Representative TEM images of mitochondria in endothelial cells from transplanted kidneys of mice from the two groups (Scale bar = 1 µm). (Green arrowheads: normal mitochondria; Red arrowhead: damaged mitochondria; Yellow arrowhead: mitophagosome). (F,G) WB results and semi‐quantitative analyses of Cyto C, BNIP3 and LC3 expression in transplanted kidney tissues of mice from the WT Allo and HIF‐1α^−/−^ Allo groups (Biological replicates, n = 3). (H,I) Representative IF double‐staining images for colocalization of CD31 with LC3 (white arrows, H) and BNIP3 (white arrows, I) in transplanted kidney tissues of mice from the WT Allo and HIF‐1α^−/−^ Allo groups (Biological replicates, n = 3. Scale bar = 20 µm). (J) Representative images of PAS and Masson staining in transplanted kidney tissues of mice from the two groups (Biological replicates, n = 3. Scale bar = 20 µm). (K) WB results of EMCN, FN, Collagen I, αSMA and CD31 expression in transplanted kidney tissues of mice from the two groups (Biological replicates, n = 3). (L) Representative IHC staining images and semi‐quantitative analyses of FN and Collagen I expression and distribution in transplanted kidney tissues of mice from the two groups (Biological replicates, n = 3. Scale bar = 20 µm). (M): The microvessel densities presented by IF staining assays for CD31 in transplanted kidney tissues of mice from different groups (Biological replicates, n = 3. Scale bar = 25 µm). Statistical significance was assessed by two‐tailed Student's *t*‐test (D, F, G, and L, M). Data were presented as mean ± SEM and *p < 0.05; **p < 0.01; ***p < 0.001; ns, not significant compared with WT Allo groups.

Evaluation of mitochondrial function revealed increased mitochondrial damage in the HIF‐1α^−/−^ Allo group by TEM analyses (Figure 11E), along with elevated Cyto C release detected by WB analyses (Figure 11F). HIF‐1α ablation also decreased LC3 and BNIP3 expression and reduced CD31 colocalization with LC3 and BNIP3, confirming suppression of BNIP3‐mediated mitophagy in renal allograft endothelial cells (Figure 11G–I). Pathological analyses demonstrated pronounced glomerular sclerosis and ECM deposition in the HIF‐1α^−/−^ Allo group (Figure 11J), accompanied by upregulation of fibrotic markers (FN, Collagen I, αSMA) and downregulation of EMCN and CD31 compared with WT Allo group, as confirmed by WB and IHC staining (Figures 11K,L and S6). In addition, microvessel densities were significantly decreased in the HIF‐1α^−/−^ Allo group compared with WT Allo group (Figure 11M). Additionally, in vitro experiments showed that BNIP3 overexpression restored mitophagy and attenuated TNFα‐induced EndMT in HIF‐1α–silenced endothelial cells (Figure S7). Collectively, these results indicate that endothelial‐specific ablation of HIF‐1α exacerbates renal allograft interstitial fibrosis by impairing BNIP3‐mediated mitophagy.

## 3 Discussion

In this study, we identified TNFα as a key inflammatory cytokine that was upregulated in the process of renal allograft interstitial fibrosis. During kidney fibrosis progression, endothelial cells exhibited pronounced mitochondrial dysfunction, EndMT and activated mitophagy. Treatment with the anti‐TNFα antibody infliximab could mitigate mitochondrial damage in endothelial cells, EndMT and renal allograft fibrogenesis, whereas mitophagy inhibition by BNIP3 knockdown exacerbated mitochondrial impairment and EndMT in endothelial cells, and aggravated renal allograft interstitial fibrosis. Mechanistically, TNFα disrupted the interaction between HIF‐1α and NEDD4, preventing proteasomal degradation of HIF‐1α. Stabilized HIF‐1α subsequently translocated to the nucleus and upregulated BNIP3 mRNA expression, activating mitophagy as a compensatory response to TNFα‐induced mitochondrial damage and ROS accumulation in endothelial cells. This mitophagic response helped restore mitochondrial function and attenuate renal allograft interstitial fibrosis. Collectively, these findings identify a TNFα–NEDD4–HIF‐1α–BNIP3 signaling module that links inflammatory stress to a compensatory mitophagy response in renal allograft endothelium, providing mechanistic insight into renal allograft interstitial fibrosis (Figure 12).

**FIGURE 12:**
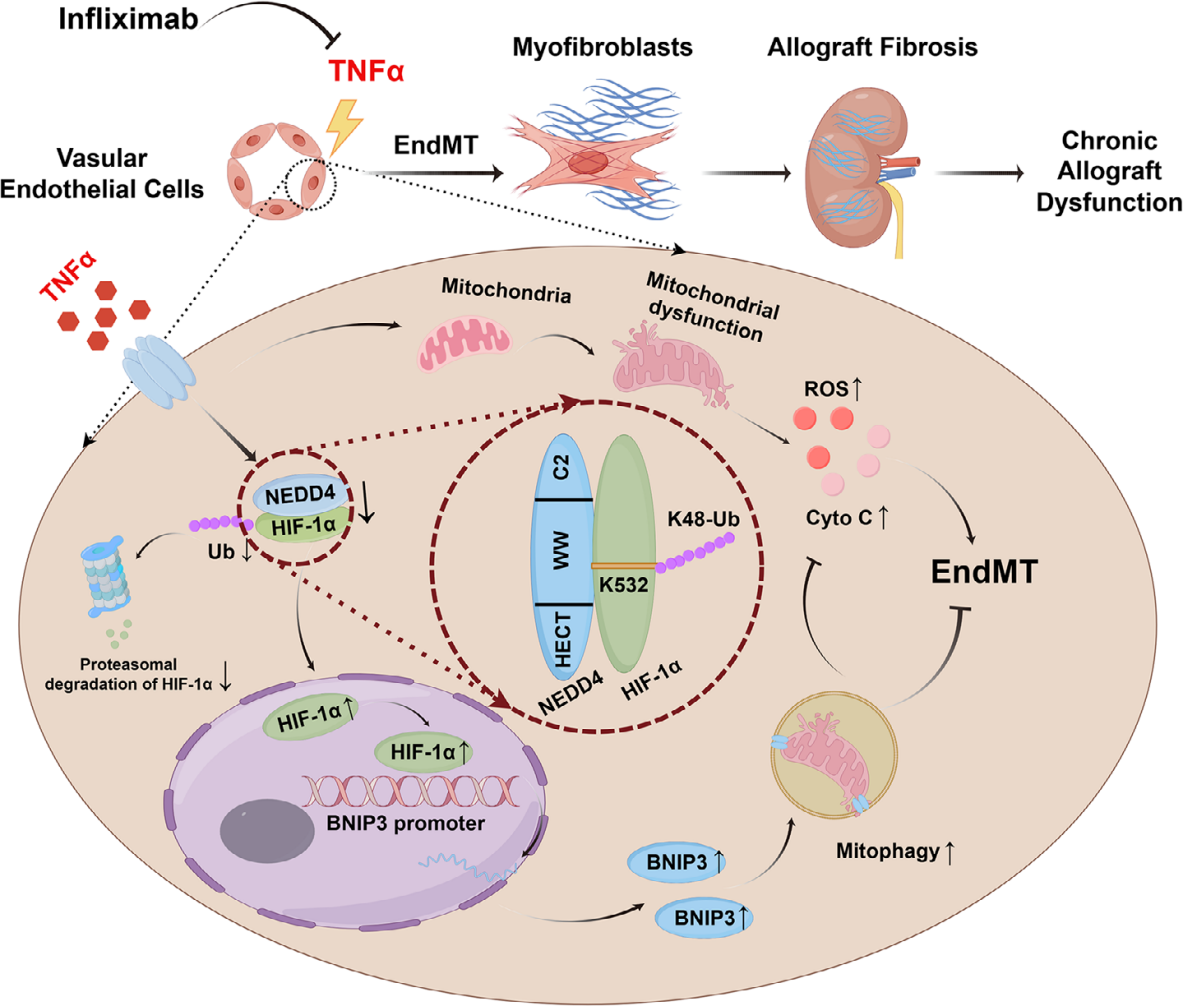
NEDD4‐HIF‐1α‐BNIP3‐mediated mitophagy alleviates endothelial‐to‐mesenchymal transition and renal allograft interstitial fibrosis induced by TNFα. The role of TNFα in the process of renal allograft interstitial fibrosis is complex and multifaceted. As it can promote renal allograft interstitial fibrosis by inducing mitochondrial dysfunction and EndMT, and also mediating compensatory mitophagy through the NEDD4–HIF‐1α–BNIP3 pathway, which clears damaged mitochondria and inhibits EndMT, thereby delaying the progression of renal allograft interstitial fibrosis.

Although progressive ECM accumulation and fibroblast activation represent convergent endpoints of kidney scarring, the upstream drivers differ between native renal fibrosis and renal allograft fibrosis [36]. In native kidneys, fibrosis is generally considered as a maladaptive repair response to repeated or sustained parenchymal injury, in which tubular epithelial injury, persistent inflammation, and microvascular rarefaction cooperate to promote interstitial myofibroblast expansion and ECM deposition, culminating in irreversible scarring and functional decline [37, 38]. By contrast, renal allograft fibrosis is a final common histologic outcome arising from multiple transplant‐specific injuries [39]. Alloimmune injury (T cell–mediated and antibody‐mediated rejection) and, particularly microvascular inflammation and endothelial injury, is a major determinant of late renal allograft dysfunction and fibrotic remodeling [39, 40]. In addition, nonimmune insults enriched in kidney transplantation—including ischemia–reperfusion injury, calcineurin‐inhibitor nephrotoxicity, viral nephropathy, recurrent renal disease, and chronic hemodynamic stress and so on—can converge on the same fibrotic phenotype [41, 42, 43]. In this context, our data suggest that TNFα‐driven inflammatory stress compromises endothelial mitochondrial homeostasis and promotes EndMT‐associated phenotypic changes, thereby facilitating fibrogenic remodeling in the renal allograft. We propose that this pathway may act as a transplant‐relevant amplification mechanism superimposed on shared downstream fibrosis programs; however, whether TNFα‐driven endothelial mitochondrial stress plays an equivalent role in native kidney fibrosis warrants further validation in non‐transplant models.

TNFα, initially identified as a key anti‐tumor cytokine [44], is now recognized as a critical profibrotic mediator in multiple fibrotic diseases [45, 46, 47]. Our previous studies demonstrated that TNFα promotes renal allograft interstitial fibrosis by facilitating epithelial‐to‐mesenchymal transition [48, 49]. Consistent with this, accumulating evidence shows that TNFα exerts profibrogenic effects through diverse pathways, including Akt and NF‐κB signaling [50, 51], and by potentiating TGF‐β/Smad transduction [52]. In the present study, we observed that the correlations in Figure 1L are unusually strong; while they support a tight association between TNFα burden and EndMT markers, correlation does not imply causality, which is further addressed by our mechanistic experiments. Then, we further revealed that TNFα is associated with vascular EndMT in vitro and renal interstitial fibrosis in renal allografts, and TNFα neutralization with infliximab could attenuate these profibrotic phenotypes in our models. Moreover, because anti‐TNFα therapy broadly modulates inflammatory networks, our in vivo data should be viewed as supportive evidence rather than definitive proof. Besides, our in vitro experiments rely on relatively high concentrations of TNFα; we therefore interpret this model as a stress‐challenge system to dissect a specific signaling axis. Accordingly, the BNIP3‐mediated mitophagy observed here likely represents a “stress‐threshold” response within a more complex chronic disease process.

Renal microvascular rarefaction is increasingly viewed as a pivotal mechanism driving the AKI‐to‐CKD transition [53]. EndMT may accelerate fibrosis not only through mesenchymal reprogramming but also by impairing endothelial integrity and capillary maintenance [54]. In the kidney transplantation setting, early microvascular loss after transplantation has been linked to later IF/TA and poorer allograft function [55]. In line with these reports, we found that EndMT coincided with reduced renal microvascular density in our model, suggesting that inflammation‐driven EndMT may contribute to chronic renal allograft fibrosis.

Mitochondrial dysfunction disrupts energy homeostasis, leading to metabolic reprogramming and bioenergetic failure in kidney cells [9]. It has been implicated in the pathogenesis of diverse kidney diseases, including senescence [17], diabetic nephropathy [56], and kidney fibrosis [57]. Several studies have explored the mechanisms underlying mitochondrial dysfunction in kidney transplantation. Clinically, reduced mitochondrial membrane potential—a marker of mitochondrial dysfunction—was associated with delayed allograft function [58]. Zhu et al. reported that elevated miR‐147 level induced mitochondrial dysfunction and renal tubular cells death by repressing NDUFA4[59], while HDAC3 inhibition preserved mitochondrial membrane potential and reduces mitochondria‐related apoptosis, thereby improving mitochondrial integrity following cold storage/rewarming injury [60]. However, these studies primarily focused on early post‐transplant stages, whereas our investigation emphasizes the relationship between mitochondrial function and renal allograft interstitial fibrosis. We observed mitochondrial damage in fibrotic transplanted kidneys, and in vascular endothelial cells, TNFα impaired mitochondrial function and increased ROS generation, promoting EndMT formation. Importantly, infliximab treatment was associated with improved mitochondrial integrity and reduced EndMT‐ and fibrosis‐related phenotype in transplanted kidneys.

Mitophagy–apoptosis crosstalk is increasingly recognized as a mitochondrial “decision node:” efficient mitophagy removes dysfunctional mitochondria and limits mtROS, thereby increasing the threshold for caspase activation, whereas overwhelming injury can shift cells toward death programs and even suppress mitophagy [61, 62]. BNIP3 sits at this interface with bidirectional outputs—it can participate in pro‐death mitochondrial signaling in endothelial injury, yet it also functions as a mitophagy receptor capable of directly initiating autophagosome biogenesis on mitochondria [63, 64]. In the kidneys, HIF‐1α–BNIP3–dependent mitophagy has been shown to mitigate fibrotic remodeling by reducing mitochondrial ROS and inflammasome activation [19]. Here, our findings support a protective role of BNIP3‐dependent mitophagy in renal allograft interstitial fibrosis. Nevertheless, our in vivo approaches are not fully cell‐type‐specific: BNIP3 regulates the stress programs beyond mitophagy. Thus, although the in vivo phenotypes are consistent with our model, they fail to establish definitive causality, which will require endothelial‐restricted genetic manipulation and rescue strategies in future studies.

In this study, we found that TNFα enhanced mitophagy by stabilizing HIF‐1α through disrupting NEDD4‐mediated proteasomal degradation, thereby promoting BNIP3 transcription. Although our data support a pro‐mitophagy role of TNFα in renal allograft endothelium, TNFα–mitophagy coupling is context‐dependent and can be bidirectional: TNFα impaired NIX‐mediated mitophagy in cortical neurons [20] and inhibited hepatocyte mitophagy via Miz1 degradation in NASH [65], yet induced PINK1‐dependent mitophagy in synovial fibroblasts in rheumatoid arthritis [21]. A plausible explanation for the pro‐mitophagy phenotype observed here is the chronic, sublethal inflammatory milieu of renal allografts, in which endothelial cells are exposed to persistent TNFα together with progressive mitochondrial ROS and injury. In such a metabolically constrained microenvironment—shaped by hypoxia/oxidative stress and limited energetic flexibility of endothelium—activation of a HIF‐1α–BNIP3 receptor‐mitophagy program may represent a compensatory stress‐adaptation response that promotes mitochondrial quality control and limits propagation of mitochondrial dysfunction. Together, these points support a context‐dependent framework for TNFα–mitophagy regulation and highlight the need for future studies to dissect the signaling networks that govern this response in renal allograft endothelium.

HIF‐1α signaling is highly cell‐type‐ and context‐dependent in kidney disease. Although HIF‐1α can promote fibrogenesis in certain renal compartments—particularly in tubular injury settings—by engaging maladaptive programs such as glycolytic remodeling and inflammatory amplification [66], epithelial phenotypic plasticity/EMT [67], and cell‐cycle stress linked to profibrogenic signaling [68], HIF‐1α has also been reported to activate mitochondrial quality control pathways under defined stress conditions. Notably, several renal injury studies—predominantly in tubular epithelial models—support that HIF‐1α could induce BNIP3‐dependent mitophagy, thereby limiting mitochondrial ROS, restraining inflammasome activation, and mitigating tissue injury and fibrosis [19, 69, 70]. In the present study, we focus on the endothelial compartment and the chronic inflammatory allograft milieu. Our data support an endothelial‐intrinsic, context‐specific model in which TNFα‐associated mitochondrial stress stabilizes HIF‐1α and engages the BNIP3‐mediated mitophagy as a compensatory response that helps preserve mitochondrial integrity. Nevertheless, we did not directly assess HIF‐1α‐dependent glycolytic flux in endothelial cells or potential paracrine effects on neighboring epithelial or interstitial cells in this study; future studies combining cell‐type‐resolved metabolic profiling and spatial approaches will be needed to define how endothelial HIF‐1α integrates into multicellular fibrotic networks.

HIF‐1α serves as a master regulator of cellular adaptation to hypoxia by coordinating metabolic reprogramming, angiogenesis, mitophagy, and cell fate decisions, exerting both protective and pathological effects depending on the context. It also acts as a pivotal transcription factor by binding the BNIP3 promoter, thereby promoting BNIP3‐mediated mitophagy [71]. In our study, TNFα promoted HIF‐1α nuclear translocation, thereby increasing BNIP3 transcriptional activity, and BNIP3 acted as a principal downstream effector mediating HIF‐1α function.

HIF‐1α function is also further modulated by posttranslational modifications, including ubiquitination, SUMOylation, and glycosylation [72, 73, 74]. Under normoxia, pVHL targets prolyl‐hydroxylated HIF‐1α for ubiquitin‐dependent degradation, maintaining appropriate hypoxic responses [75]. Beyond this classical E3 ligase, HIF‐1α is also regulated by OTUB1, USP38, and NEDD4L [76, 77, 78]. Notably, our data suggest that NEDD4 can promote proteasomal degradation of HIF‐1α protein via a WW domain–dependent interaction and K48‐linked ubiquitination at Lys532. Although TNFα clearly diminishes the HIF‐1α–NEDD4 association, it does so without detectable changes in NEDD4 abundance, phosphorylation, or localization, suggesting an indirect regulatory mechanism that remains to be elucidated.

A limitation of this study is that, in the absence of endothelial lineage tracing/fate mapping, marker‐based EndMT evidence cannot distinguish stable endothelial‐to‐myofibroblast conversion from partial or stress‐induced endothelial plasticity. Notably, the existence and quantitative contribution of EndMT to renal myofibroblast pools remain controversial in the kidney fibrosis field. Earlier marker co‐expression studies reported that EndMT could contribute to renal fibroblast accumulation in several fibrosis models (e.g., detection of CD31–positive cells co‐expressing FSP1 or αSMA) [79]. However, such estimates inherently depend on marker panels and are susceptible to both underestimation (loss of endothelial identity) and overinterpretation (partial/transient mesenchymal activation). Moreover, recent systematic fate‐mapping analyses suggest that endothelial cells contribute minimally to the pool of αSMA‐positive, ECM‐producing myofibroblasts in UUO, with resident fibroblasts as the dominant source [80]. Therefore, future studies implementing validated, endothelial‐restricted inducible fate mapping in the kidney transplant model will be necessary to quantify endothelial contribution to ECM‐producing populations.

Besides, we acknowledge several limitations. First, longitudinal assessments of kidney function and allograft perfusion will be important to strengthen the clinical relevance of our findings. Second, further mechanistic validation is warranted, including biophysical quantification of interaction kinetics/affinity and an in vivo causal rescue using the K532R mutant in the transplant model.

In summary, our study demonstrates that the role of TNFα in the progression of renal allograft interstitial fibrosis is complex and multifaceted. In endothelial cells, TNFα functions as a pivotal inflammatory mediator that drives mitochondrial dysfunction and EndMT, thereby promoting renal allograft interstitial fibrosis. Meanwhile, TNFα‐induced compensatory mitophagy via the NEDD4/HIF‐1α/BNIP3 pathway eliminates damaged mitochondria, mitigates EndMT, and alleviates renal allograft interstitial fibrosis. Overall, these findings provide mechanistic insight and a conceptual framework for future therapeutic exploration.

## 4 Methods

### 4.1 Patients and Tissue Samples

Our study followed the guiding principles of the Declaration of Helsinki and was approved by the Jiangsu Province Hospital Ethics Committee. A total of 20 kidney transplant recipients were enrolled at Jiangsu Province Hospital. CAD was diagnosed by renal biopsy or nephrectomy specimens showing pathological evidence of allograft interstitial fibrosis. Normal kidney tissues were obtained from nephrectomy specimens, at least 5 cm from the tumor margin. The studies involving human participants were approved by the Ethics Committee of the First Affiliated Hospital of Nanjing Medical University (approval number: 2023‐SRFA‐007). Informed consent was provided by all participants. The baseline characteristics of participants are presented in Table 1.

**TABLE 1:** Baseline characteristics of the Normal and CAD groups.

Clinical variables	Normal Group	CAD group	*p* value
Case number (n)	20	20	> 0.05
Gender (Male/Female)	14/6	12/8	> 0.05
Age (years, mean ± SEM)	65.15 ± 1.36	59.60 ± 2.44	> 0.05
BMI (kg/m^2^, mean ± SEM)	22.77 ± 0.36	23.51 ± 0.38	> 0.05
Transplant duration (years, mean ± SEM)	—	9.76 ± 0.42	—
Primary/secondary transplant	—	20/0	—
PRA before renal transplant (%)	—	0	—
Donor source			
Living‐related	—	4	—
Cadaveric	—	16	—
Immunosuppressive regimen			
Prednisone + MMF + Tac	—	15	—
Prednisone + MMF + CsA	—	5	—
Serum creatinine (µmol/L, mean ± SEM)	70.55 ± 3.04	435.40 ± 8.43	< 0.001

BMI, body mass index; PRA, panel reaction antibody; MMF, mycophenolate mofetil; CsA, cyclosporine A; Tac, tacrolimus. Statistical significance was assessed by two‐tailed Student's *t*‐test.

### 4.2 Animals

*HIF‐1α^flox/flox^* mice and *Tie2‐Cre* mice (C57BL/6 background) were purchased from GemPharmatech (Nanjing, China, No. T007239). *HIF‐1α^flox/flox^* mice were crossed with *Tie2‐Cre* mice to generate endothelial cell‐specific HIF‐1α knockout offspring (Genotype: *HIF‐1α* ^fl/fl^, *Cre^+/−^*). Littermates with the genotype *HIF‐1α* ^fl/fl^, *Cre^−/−^* served as controls. The primers for genotyping were as follows:

HIF‐1α genotyping: sense:5′‐GCACCTGGAAGTAGTAGCTTGTTACAGC‐3′
anti‐sense: 5′‐TTCATCCCCAAATTTACAGTAGCTTATG‐3′
Tie2‐Cre genotyping, sense: 5′‐ATTTGCCTGCATTACCGGTC‐3′
anti‐sense: 5′‐ATCAACGTTTTCTTTTCGG‐3′

Additionally, C57BL/6 and BALB/c mice were obtained from the Animal Center of Nanjing Medical University. All animals were housed in the specific pathogen‐free Laboratory Animal Center at Nanjing Medical University.

### 4.3 Mouse Models of Renal Allogeneic Transplantation

All animal experiments were approved by the Animal Research Ethics Committee of Nanjing Medical University (approval number: IACUC‐2109025). The specific modeling methods for constructing renal allogeneic transplantation mouse models were previously described [24]. In the Syn group, BALB/c mice received kidneys from BALB/c mice, while in the Allo group, C57BL/6 mice received kidneys from BALB/c mice.

Likewise, BALB/c mice receiving donor kidneys from *HIF‐1α* ^fl/fl^, *Cre^+/−^* or *HIF‐1α* ^fl/fl^, *Cre^−/−^* C57BL/6 mice were designated as the HIF‐1α^−/−^ Allo group or the WT Allo group, respectively. 2 weeks after the resection of the left native kidneys in recipient mice, tamoxifen (cat: T5648, Sigma‐Aldrich) was intraperitoneally injected for 5 days at 60 mg/kg to induce *HIF‐1α* gene ablation.

To investigate the impact of TNFα on interstitial fibrosis, the mice in the Syn and Allo groups received intraperitoneal injections of the TNFα specific inhibitor Infliximab (MCE, HY‐P9970) at a dose of 10 µg/g in 100 µl saline every 4 weeks after kidney transplantation.

### 4.4 Adeno‐Associated Virus Infection In Vivo

To induce BNIP3 depletion in vivo, AAV9 vectors carrying a control short hairpin RNA (shRNA) (AAV‐shNC) or a shBNIP3 (AAV‐shBNIP3) were constructed by Jiangsu KeyGEN BioTECH. Each mouse was injected with the AAVs (1 × 10^12^ viral genomes) via tail vein, 3 weeks after kidney transplantation.

### 4.5 Cell Cultures and Treatment

HUVECs were cultured as previously described [24]. After seeding on appropriate culture plates overnight, HUVECs were exposed to 100 ng/mL TNFα (Novoprotein, C008) for different time periods.

### 4.6 Plasmids, siRNA, and Reagents

The HA‐UB‐WT (P0554), HA‐UB‐K48 (P31802), HA‐UB‐K63 (P31800), HA‐UB‐K48R (P71870), and Flag‐HIF‐1α (P73021) plasmids were obtained from MIAOLING BIOLOGY. Flag‐tagged HIF‐1α mutations (K32R, K363R, K477R, K532R, K538R, K547R) were generated by Jiangsu KeyGEN BioTECH. HA‐BNIP3, His‐tagged NEDD4, HA‐tagged NEDD4, and HA‐tagged NEDD4 domain‐deletion plasmid were purchased from Beijing Tsingke Biotech Company.

siRNAs were constructed by Jiangsu KeyGEN BioTECH. All the transfections were performed with Lipofectamine 3000 (Invitrogen, L3000008). The siRNA sequences used are listed in Table S2.

The reagents used in this study are listed in Table S1.

### 4.7 TEM Analysis

Renal tissues and HUVECs prepared for TEM analysis were prepared as previously described [48]. The processed samples were examined using a transmission electron microscope (FEI Tecnai G2).

### 4.8 Detection of Mitochondrial Membrane Potential and ROS Level

To detect mitochondrial membrane potential, HUVECs were incubated with JC‐1 dye (Beyotime, C2003S) and analyzed by flow cytometry on a Cytoflex S (Beckman). Mitochondria‐derived ROS was detected using the mitochondrial superoxide indicator MitoSOX (Invitrogen, M36008). Briefly, HUVECs were incubated with 5 µM MitoSOX for 10 min at 37°C, subsequently analyzed by flow cytometry or an Olympus optical microscope. The ROS levels in renal tissues were determined by DHE staining (Beyotime, S0063). Frozen transplanted kidney sections were incubated with 5 µM DHE at 37°C for 30 min, and images were captured using an Olympus optical microscope.

### 4.9 Mitophagic Flux Analysis

HUVECs were infected with mitochondria‐targeted Keima (mt‐Keima) adenovirus (GeneChem) for 6 h. After infection, the medium was replaced with fresh complete culture medium, and cells were incubated for an additional 48 h to allow mKeima expression. mKeima fluorescence was then imaged using a Zeiss LSM780 confocal microscope. Because Keima exhibits a pH‐dependent excitation shift, mitochondria localized in neutral compartments and acidic lysosomes can be distinguished by sequential excitation at 488 and 561 nm, respectively. Paired images for ratiometric analysis were acquired sequentially under 488 (neutral signal) and 561 nm (acidic/lysosomal signal) excitation using identical acquisition settings across all groups. Mitophagic flux was quantified in ImageJ by calculating the fluorescence integrated density ratio (561/488 nm) for each field. For each condition, images from multiple randomly selected fields were analyzed per experiment, and at least three independent experiments were performed.

### 4.10 Seahorse Extracellular Flux Analysis (OCR and ECAR)

OCR and ECAR were measured using a Seahorse XF analyzer (Agilent). HUVECs subjected to the indicated transfections and TNFα treatment were seeded in Seahorse microplates and equilibrated in Seahorse XF Base Medium (pH 7.4) before the assay. For the Mito Stress Test, OCR was recorded at baseline and after sequential injections of oligomycin (2 µM; MCE, HY‐N6782), FCCP (1 µM; MCE, HY‐100410), and rotenone/antimycin A (Rot/AA) (1 µM; MCE, HY‐B1756). For the Glycolysis Stress Test, ECAR was recorded at baseline and after sequential injections of Rot/AA (1 µM) and 2‐deoxy‐D‐glucose (2‐DG) (50 mM; MCE, HY‐13966). Parameters were calculated using Seahorse Wave software. Data were normalized to cell number or total protein.

### 4.11 Mitochondrial Mass Measurement

Mitochondrial mass was assessed by staining live cells with MitoTracker Green FM (Thermo Fisher Scientific, M46750) at 200 nM for 30 min at 37°C. After washing, fluorescence was quantified by flow cytometry under identical acquisition settings. Data were expressed as mean fluorescence intensity per cell. Flow cytometry data were analyzed using FlowJo software (v10, BD Biosciences).

### 4.12 RNA Extraction and Reverse‐Transcriptase PCR Assay

Total cellular RNA was isolated, followed by reverse transcription using the SuperMix (Vazyme, R222‐01). Quantitative PCR was performed on the StepOnePlus Real‐Time PCR system (Applied Biosystems) using the ChamQ SYBR qPCR Master Mix (Vazyme, Q341‐02). The specific primer sequences were listed in Table S2.

### 4.13 WB and Co‐IP Assays

Briefly, for WB assays, proteins were extracted from cells or tissues, then quantified, separated and transferred to PVDF membranes. After blocking using a 5% nonfat milk, the membranes were incubated with primary antibodies and secondary antibodies, and then detected with ECL reagent.

For Co‐IP assays, protein samples were obtained from different cell groups, and then incubated with primary antibodies and Protein A/G magnetic beads to form the protein‐bead complexes. Subsequently, the proteins were separated from the complexes and subjected to WB analysis.

The antibodies used are listed in Table S1.

### 4.14 CHX Chase Assays

The half‐lives of proteins were determined by CHX chase assays. Briefly, cells from different groups were treated with CHX (200 µg/mL) for various time intervals, and then lysed for WB analyses.

### 4.15 Dual Luciferase Reporter Assay

The predicted BNIP3 promoter reporter plasmid (pGL3.0‐BNIP3) was generated by Tsingke Biotechnology. Control or HIF‐1α siRNA was co‐transfected with the pGL3.0‐BNIP3 plasmids (+1868 to +1873) using Lipofectamine 3000 (Invitrogen, L3000008). After 48 h of co‐transfection, dual luciferase assays (Vazyme, DL101‐01) were conducted according to the manufacturer's instructions.

### 4.16 ChIP Assay

ChIP assay was performed with the ChIP Assay kit (Beyotime, P2083S) following the manufacturer's protocol. DNA was sonicated into fragments ranging from 100 and 1000 bp using a sonicator. Subsequently, equal amounts of DNA were incubated with either an HIF‐1α antibody or an IgG‐negative control, along with 20 µL magnetic protein G beads overnight at 4°C. Following de‐crosslinking and proteinase treatment, the purified DNA was collected and subjected to ChIP‐qPCR. ChIP signals were normalized to input DNA and expressed as % input, with a negative control region included as an internal negative control.

### 4.17 Histopathological Staining of Renal Allografts

Histological analyses of renal allografts were conducted using PAS staining and Masson trichrome staining. Masson staining was employed to evaluate the degree of renal interstitial fibrosis, while PAS staining was used to evaluate changes in allograft structure.

### 4.18 IHC and Indirect IF Staining Assays

IHC staining was performed using the HRP Kit (CWbio, CW2069S). For IF staining, renal sections were deparaffinized, rehydrated, retrieved, blocked, and then incubated with the primary and secondary antibodies.

For cell IF staining, cell samples were fixed, permeabilized, blocked, and then incubated with the primary and secondary antibodies. MitoTracker Red CMXRos (Invitrogen, M7512) was used for labeling the mitochondria. Images were captured using either a Zeiss LSM 880 NLO confocal microscope or an Olympus optical microscope.

The antibodies used are listed in Table S1.

### 4.19 Enzyme‐Linked Immunosorbent Assay (ELISA)

Serum levels of TNFα, IL‐1β, and IL‐6 were quantified across different groups using ELISA kits (Elabscience; Cat. Nos. M3063, H0109, H0149, and H6156).

### 4.20 RNA Stability Assay

HUVECs were seeded in 6‐well plates and pretreated with or without TNFα treatment for 24 h. Transcription was then inhibited by actinomycin D (MedChemExpress, HY‐17559; 5 µg/ml), and cells were harvested at 0, 2, 4, and 6 h after actinomycin D treatment. Total RNA was extracted and BNIP3 mRNA was quantified by RT–qPCR.

### 4.21 Statistical Analysis

Data are presented as mean ± standard error of the mean (SEM), unless otherwise indicated. The sample size (n) for each experiment is reported in the corresponding figure legends and represents independent biological replicates per group (for in vivo experiments, one animal corresponds to one biological replicate). Normality was assessed where sample size permitted. For comparisons between two groups, two‐tailed Student's t‐test was used. For experiments with more than two groups, one‐way ANOVA followed by Dunnett's post hoc test (vs. a single control) or two‐way ANOVA followed by Sidak's post hoc test (for multiple pairwise comparisons) was applied as appropriate. Statistical significance was defined as *p* < 0.05, or *q* < 0.05 for FDR‐adjusted analyses. Correlation analyses were performed using Pearson correlation, with simple linear regression shown where indicated. All analyses were conducted using GraphPad Prism (version 10.0).

## Author Contributions

Ruoyun Tan, Jianjian Zhang and Min Gu designed the study; Dengyuan Feng, Qinghuan Shen, Junqi Zhang, and Jiawen Liu performed the experiments; Dengyuan Feng, Qinghuan Shen, Jiawen Liu, Jianjian Zhang, Zeping Gui, Zijie Wang, Qianguang Han, Shuang Fei, Hao Chen, Li Sun, Jun Tao, Zhijian Han, Xiaobing Ju and Ruoyun Tan acquired and analyzed the data; Dengyuan Feng wrote the paper; Ruoyun Tan, Jianjian Zhang and Min Gu revised the manuscript. All authors read and approved the final manuscript.

## Conflicts of Interest

The authors declare no conflicts of interest.

## Supporting information




**Supporting File 1**: advs76746‐sup‐0001‐FigureS1‐S7.docx.

**Supporting File 2**: advs76746‐sup‐0002‐TableS1‐S3.zip.

## Data Availability

All relevant data are available in the figures and materials. Any additional information required is available from the corresponding authors.
