# Supplementary material for: Compensatory Mitophagy via NEDD4/HIF‐1α/BNIP3 Pathway Restrains EndMT and Renal Allograft Interstitial Fibrosis Induced by TNFα
Source: Adv Sci (Weinh). 2026 Sep 20;13(58):e16953. doi: 10.1002/advs.202516953 (PMC13590865; doi:10.1002/advs.202516953)
Supplement: Supplementary file 1 [file ADVS-13-e16953-s001.docx]

**Supplementary Figure 1:** Patients and renal tissues information for single-cell RNA sequencing (GSE195718), including normal renal allograft tissues from three stable transplant recipients (Normal allograft group) and fibrotic renal allograft tissues from six patients with chronic allograft dysfunction (Fibrotic allograft group).

**Supplementary Figure 2: A:** Semi**-**quantitative analyses of Western blot results in Figure 4A for EMCN, CD31, FN, and αSMA protein expression in HUVECs treated with different concentrations of TNFα (Biological replicate, n = 3). **B**: Semi-quantitative analyses of IF staining assays in Figure 4B for CD31 and FN expression in HUVECs treated with different concentrations of TNFα (Biological replicate, n = 4. Scale bar = 20μm). **C**: Semi-quantitative analyses of Western blot results in Figure 4D for Cyto C and Cleaved Caspase 3 expression in HUVECs treated with different concentrations of TNFα (Biological replicate, n = 3). **D**: Quantitative analyses of TEM images in Figure 4E in control and TNFα-treated HUVECs (Biological replicate, n = 6. Scale bar = 1μm). **E**: Quantitative analyses of MitoTracker Red staining assays in Figure 4F in HUVECs treated with different concentrations of TNFα, analyzed using the Mitochondria Analyzer plugin for ImageJ (Biological replicate, n = 11. Scale bar = 5μm). **F**: Quantitative analyses of flow cytometry assays for JC-1 staining in Figure 4H in HUVECs treated with different concentrations of TNFα (Biological replicate, n = 3). **G**: Semi-quantitative analyses of Western blot results in Figure 4J for TOMM20, LC3, p-ULK1, and ATG5 expression in HUVECs treated with different concentrations of TNFα (Biological replicate, n = 3). **H**: Quantitative analyses of TEM images in Figure 4K in control and TNFα-treated HUVECs (Biological replicate, n = 6. Scale bar = 1μm). **I**: Quantitative analyses of mt-Keima mitophagy signals in Figure 4M in different groups (Biological replicate, n = 8. Scale bar = 10μm). **J**: Semi-quantitative analyses of Western blot results in Figure 4N for PINK1, NIX, FUNDC1, and BNIP3 expression in HUVECs treated with varying concentrations of TNFα (n = 3). **K**: Quantitative analyses of IF staining assays in Figure 4O for MitoTracker and BNIP3 in HUVECs treated with different concentrations of TNFα (Biological replicate, n = 5. Scale bar = 5μm). Statistical significances were assessed by two-tailed Student’s *t*-test (D and H), one-way ANOVA with Dunnett’s post hoc test (A-C, E-G, and J-K), and two-way ANOVA with Sidak’s post hoc test (I). Data were presented as mean ± SEM and **p* < 0.05; ***p* < 0.01; ****p* < 0.001; ns, not significant compared to control group.

**Supplementary Figure 3:** Relative mRNA expression levels of BNIP3 in control and TNFα-treated HUVECs at 0, 2, 4, and 6 h after actinomycin D treatment (n=3). Statistical significance was assessed by two-way ANOVA with Sidak’s post hoc test. Data were presented as mean ± SEM and ns: difference not significant compared to control group.

**Supplementary Figure 4:** KEGG pathway analysis identified the transcription factors that could regulate BNIP3 expression (Red box).

**Supplementary Figure 5:** Western blot results and quantitative analyses of P65 and phosphorylated P65 (p-P65) expression in HUVECs treated with TNFα for various time points (n = 3). Statistical significance was assessed by two-way ANOVA with Sidak’s post hoc test. Data were presented as mean ± SEM and ***p* < 0.01; ****p* < 0.001; ns, not significant compared to control group.

**Supplementary Figure 6:** Quantitative analyses of Western blot results in Figure 11K for EMCN, FN, Collagen I, αSMA and CD31 expression in transplanted kidney tissues from the two groups (n = 3). Statistical significance was assessed by two-way ANOVA with Sidak’s post hoc test. Data were presented as mean ± SEM and **p* < 0.05; ***p* < 0.01; ****p* < 0.001, compared to WT Allo group.

**Supplementary Figure 7:** BNIP3 over-expression restores mitophagy and attenuates TNFα-induced EndMT in HIF-1α–silenced endothelial cells.

HUVECs were transfected with siNC or siHIF-1α for 24 h, then transfected with empty vector or a BNIP3 expression plasmid for an additional 24 h, followed by TNFα treatment for 24 h. **A, B**: Western blot results and semi-quantitative analyses of BNIP3, ATG5, LC3, CD31, FN, and αSMA expression from different groups (Biological replicate, n = 3). Statistical significance was assessed by two-way ANOVA with Sidak’s post hoc test (A and B). Data were presented as mean ± SEM and **p* < 0.05; ***p* < 0.01; ****p* < 0.001, compared to Vetor+TNFα or siHIF-1α+TNFα group.
