# Supplementary material for: Compensatory Mitophagy via NEDD4/HIF‐1α/BNIP3 Pathway Restrains EndMT and Renal Allograft Interstitial Fibrosis Induced by TNFα
Source: Adv Sci (Weinh). 2026 Sep 20;13(58):e16953. doi: 10.1002/advs.202516953 (PMC13590865; doi:10.1002/advs.202516953)
Supplement: Supplementary file 2 [file ADVS-13-e16953-s002.zip › Supplementary Table 1.docx]

**Supplementary Table 1: The antibodies and reagents used in this study.**

| **Antibodies for western blotting** | **Company** | **Identifier** |
| --- | --- | --- |
| TNFα | Cell Signaling Technology | #11948 |
| CD31 | Cell Signaling Technology | #77699 |
| Fibronectin | Proteintech | 15613-1-AP |
| Collagen І | ABclonal | A1352 |
| αSMA | Cell Signaling Technology | #19245 |
| LC3 | Cell Signaling Technology | #3868 |
| TOMM20 | Proteintech | 66777-1-Ig |
| Cleaved Caspase 3 | Proteintech | 25128-1-AP |
| Cytochrome c | Proteintech | 10993-1-AP |
| PINK1 | Cell Signaling Technology | #6946 |
| NIX | Cell Signaling Technology | #12396 |
| FUNDC1 | ABclonal | A16318 |
| BNIP3 | Cell Signaling Technology | #44060 |
| BNIP3 | Proteintech | 68091-1-Ig |
| P65 | Cell Signaling Technology | #8242 |
| p-P65 | Cell Signaling Technology | #3033 |
| HIF-1α | Proteintech | 20960-1-AP |
| VHL | Proteintech | 24756-1-AP |
| Ubiquitin | Santa Cruz | sc-8017 |
| β-ACTIN | Proteintech | 66009-1-Ig |
| Goat Anti-Mouse IgG | Proteintech | SA00001-1 |
| Goat Anti-Rabbit IgG | Proteintech | SA00001-2 |

| **Antibodies for immunofluorescence** | **Company** | **Identifier** |
| --- | --- | --- |
| CD31 | R&D Systems | AF3628 |
| Rictor | Abcam | ab104838 |
| BNIP3 | Cell Signaling Technology | #44060 |
| FN | Proteintech | 15613-1-AP |
| HIF-1α | Proteintech | 20960-1-AP |
| VHL | Proteintech | 24756-1-AP |
| LC3 | Cell Signaling Technology | #3868 |
| Cy™3 AffiniPure Goat Anti-Rabbit IgG | Jackson ImmunoResearch | 111-165-003 |
| Fluorescein (FITC) AffiniPure Goat Anti-Mouse IgG | Jackson ImmunoResearch | 115-095-003 |
| Cy3-labeled Donkey Anti-Goat IgG | Beyotime | A0502 |
| Fluorescein (FITC) AffiniPure Donkey Anti-Rabit IgG (H+L) | Jackson ImmunoResearch | 711-095-152 |
| **reagents** | **Company** | **Identifier** |
| TNFα | Novoprotein | C008 |
| MG132 | Selleck | S2619 |
| CHX | Selleck | S7418 |
| CQ | Selleck | S6999 |
| Actinomycin D | MedChemExpress | HY-17559 |
