# Supplementary material for: Compensatory Mitophagy via NEDD4/HIF‐1α/BNIP3 Pathway Restrains EndMT and Renal Allograft Interstitial Fibrosis Induced by TNFα
Source: Adv Sci (Weinh). 2026 Sep 20;13(58):e16953. doi: 10.1002/advs.202516953 (PMC13590865; doi:10.1002/advs.202516953)
Supplement: Supplementary file 2 [file ADVS-13-e16953-s002.zip › Supplementary Table 2.docx]

**Supplementary Table 2: The** **siRNA sequences and specific primer sequences used in this study.**

| **siRNA** | **Sequences** |
| --- | --- |
| BNIP3 | 5′‐GAACUGCACUUCAGCAAUAAUTT‐3′ |
| HIF-1α | 5′-CUGAUGACCAGCAACUUGA-3′ |

| **Genes** | **Primer sequences** |
| --- | --- |
| BNIP3 | Forward: 5′-CAGGGCTCCTGGGTAGAACT-3′ |
|  | Reverse: 5′-CTACTCCGTCCAGACTCATGC-3′ |
| HIF-1α | Forward: 5′-GAACGTCGAAAAGAAAAGTCTCG-3′ |
|  | Reverse: 5′-CCTTATCAAGATGCGAACTCACA-3′ |
| GAPDH | Forward: 5′-GGAGCGAGATCCCTCCAAAAT-3′ |
|  | Reverse: 5′- GGCTGTTGTCATACTTCTCATGG-3′ |
