# Supplementary material for: Compensatory Mitophagy via NEDD4/HIF‐1α/BNIP3 Pathway Restrains EndMT and Renal Allograft Interstitial Fibrosis Induced by TNFα
Source: Adv Sci (Weinh). 2026 Sep 20;13(58):e16953. doi: 10.1002/advs.202516953 (PMC13590865; doi:10.1002/advs.202516953)
Supplement: Supplementary file 2 [file ADVS-13-e16953-s002.zip › Supplementary Table 3.docx]

**Supplementary Table 3: The potential binding sites between HIF-1α and the BNIP3 promoter region predicted using the JASPAR database.**

| **Matrix ID** | **Name** | **Score** | **Relative score** | **Sequence ID** | **Start** | **End** | **Strand** | **Predicted sequence** |
| --- | --- | --- | --- | --- | --- | --- | --- | --- |
| **MA1106.2** | MA1106.2.HIF1A | 11.279793 | 0.9999999876708631 | NC_000010.11:c131983923-131981824 | 1868 | 1873 | + | ACGTGC |
| **MA1106.2** | MA1106.2.HIF1A | 11.279793 | 0.9999999876708631 | NC_000010.11:c131983923-131981824 | 1502 | 1507 | - | ACGTGC |
| **MA1106.2** | MA1106.2.HIF1A | 11.279793 | 0.9999999876708631 | NC_000010.11:c131983923-131981824 | 1866 | 1871 | - | ACGTGC |
